# Comparison of RNA-seq and microarray-based models for clinical endpoint prediction

**DOI:** 10.1186/s13059-015-0694-1

**Published:** 2015-06-25

**Authors:** Wenqian Zhang, Ying Yu, Falk Hertwig, Jean Thierry-Mieg, Wenwei Zhang, Danielle Thierry-Mieg, Jian Wang, Cesare Furlanello, Viswanath Devanarayan, Jie Cheng, Youping Deng, Barbara Hero, Huixiao Hong, Meiwen Jia, Li Li, Simon M Lin, Yuri Nikolsky, André Oberthuer, Tao Qing, Zhenqiang Su, Ruth Volland, Charles Wang, May D. Wang, Junmei Ai, Davide Albanese, Shahab Asgharzadeh, Smadar Avigad, Wenjun Bao, Marina Bessarabova, Murray H. Brilliant, Benedikt Brors, Marco Chierici, Tzu-Ming Chu, Jibin Zhang, Richard G. Grundy, Min Max He, Scott Hebbring, Howard L. Kaufman, Samir Lababidi, Lee J. Lancashire, Yan Li, Xin X. Lu, Heng Luo, Xiwen Ma, Baitang Ning, Rosa Noguera, Martin Peifer, John H. Phan, Frederik Roels, Carolina Rosswog, Susan Shao, Jie Shen, Jessica Theissen, Gian Paolo Tonini, Jo Vandesompele, Po-Yen Wu, Wenzhong Xiao, Joshua Xu, Weihong Xu, Jiekun Xuan, Yong Yang, Zhan Ye, Zirui Dong, Ke K. Zhang, Ye Yin, Chen Zhao, Yuanting Zheng, Russell D. Wolfinger, Tieliu Shi, Linda H. Malkas, Frank Berthold, Jun Wang, Weida Tong, Leming Shi, Zhiyu Peng, Matthias Fischer

**Affiliations:** BGI-Shenzhen, Main Building, Bei Shan Industrial Zone, Yantian District, Shenzhen, Guangdong 518083 China; Collaborative Innovation Center for Genetics and Development, State Key Laboratory of Genetic Engineering and MOE Key Laboratory of Contemporary Anthropology, School of Life Sciences and School of Pharmacy, Fudan University, Shanghai, 201203 China; Department of Pediatric Oncology and Hematology, University Children’s Hospital of Cologne, Kerpener Strasse 62, D-50924 Cologne, Germany; University of Cologne, Center for Molecular Medicine (CMMC), Medical Faculty, Kerpener Strasse 62, D-50924 Cologne, Germany; NIH/NCBI, Bldg 38A/Room 8S808, 8600 Rockville Pike, Bethesda, MD 20894 USA; Eli Lilly and Company Research Informatics, Lilly Corporate Center, Drop Code 0725, Indianapolis, IN 46285 USA; Fondazione Bruno Kessler (FBK), Via Sommarive 18, 38123 Trento Povo, TN Italy; AbbVie Inc., Global Pharmaceutical R&D, 32 Knights Crest Court, Souderton, PA 18964 USA; GlaxoSmithKline, Discovery Analytics, Mailstop UP4335, 1250 South Collegeville Rd, Collegeville, PA 19426 USA; Department of Internal Medicine, Rush University Cancer Center, 1725 W. Harrison Street, Chicago, IL 60612 USA; National Center for Toxicological Research, U.S. Food and Drug Administration, 3900 NCTR Road, Jefferson, AR 72079 USA; SAS Institute Inc., SAS Campus Drive, Cary, NC 27513 USA; Marshfield Clinic Research Foundation, Biomedical Informatics Research Center, 1000 N Oak Avenue, Marshfield, WI 54449 USA; Thomson Reuters IP & Science, 5901 Priesty Drive, Carlsbad, CA 92008 USA; Center for Genomics and Division of Microbiology & Molecular Genetics, School of Medicine, Loma Linda University, Loma Linda, CA 92350 USA; Department of Biomedical Engineering, GeorgiaTech and Emory University, 313 Ferst Drive, Atlanta, GA 30332 USA; Fondazione Edmund Mach, CRI-CBC, San Michele all’Adige, TN Italy; Children’s Hospital Los Angeles, Los Angeles, CA 90027 USA; Department of Pediatric Hematology-Oncology, Molecular Oncology, Felsenstein Medical Research Center, Schneider Children’s Medical Center of Israel, Petach Tikva, 49202 Israel; Marshfield Clinic Research Foundation, Center of Human Genetics, 1000 N Oak Avenue, Marshfield, WI 54449 USA; Department of Theoretical Bioinformatics, German Cancer Research Center (DKFZ), Im Neuenheimer Feld 280, D-69120 Heidelberg, Germany; University of Nottingham, Children’s Brain Tumour Research Centre, Queen’s Medical Centre, University of Nottingham, D Floor Medical School, Nottingham, NG7 2UH UK; Center for Biologics Evaluation and Research, U.S. Food and Drug Administration, WOC1 RM400S, HFM-210, 1401 Rockville Pike, Rockville, MD 20852 USA; AbbVie Inc., Global Pharmaceutical Research and Development, 1 North Waukegan Road, North Chicago, IL 60064 USA; University of Arkansas at Little Rock, UALR/UAMS Joint Bioinformatics Graduate Program, 2801 South University Avenue, Little Rock, AR 72204 USA; Eli Lilly and Company, Discovery Statistics, Lilly Corporate Center, Drop Code 2036, Indianapolis, IN 46285 USA; Department of Pathology, University of Valencia, Medical School, Avda. Blasco Ibáñez, 17, 46010 Valencia, Spain; Department of Translational Genomics, University of Cologne, D-50924 Cologne, Germany; Neuroblastoma Laboratory, Onco/Hematology Laboratory, SDB Department, University of Padua, Pediatric Research Institute, Padua, Italy; Department of Pediatrics and Genetics, Ghent University, Center for Medical Genetics, Ghent University, De Pintelaan 185, 9000 Ghent, Belgium; Georgia Institute of Technology, School of Electrical and Computer Engineering, 777 Atlantic Drive NW, Atlanta, GA 30332 USA; Harvard Medical School, Massachusetts General Hospital, 51 Blossom Street, Boston, MA 02114 USA; Stanford University, Stanford Genome Technology Center, 855 South California Avenue, Palo Alto, CA 94304 USA; Department of Pathology, University of North Dakota School of Medicine, 501 N. Columbia Road RM 3573, Grand Forks, ND 58202-9037 USA; East China Normal University, Center for Bioinformatics and Computational Biology, Shanghai Key Laboratory of Regulatory Biology, the Institute of Biomedical Sciences and School of Life Sciences, 500 Dongchuan Road, Shanghai, 200241 China; Department of Molecular & Cellular Biology, Beckman Research Institute, City of Hope Comprehensive Cancer Center, Duarte, CA 91010 USA; Department of Biology, University of Copenhagen, Copenhagen, DK-2200 Denmark; King Abdulaziz University, Jeddah, 21589 Saudi Arabia; Novo Nordisk Foundation Center for Basic Metabolic Research, University of Copenhagen, Copenhagen, DK-2200 Denmark; BGI-Guangzhou, Guangzhou Higher Education Mega Center, No. 280, Waihuan East Rd., Guangzhou, 510006 China

## Abstract

**Background:**

Gene expression profiling is being widely applied in cancer research to identify biomarkers for clinical endpoint prediction. Since RNA-seq provides a powerful tool for transcriptome-based applications beyond the limitations of microarrays, we sought to systematically evaluate the performance of RNA-seq-based and microarray-based classifiers in this MAQC-III/SEQC study for clinical endpoint prediction using neuroblastoma as a model.

**Results:**

We generate gene expression profiles from 498 primary neuroblastomas using both RNA-seq and 44 k microarrays. Characterization of the neuroblastoma transcriptome by RNA-seq reveals that more than 48,000 genes and 200,000 transcripts are being expressed in this malignancy. We also find that RNA-seq provides much more detailed information on specific transcript expression patterns in clinico-genetic neuroblastoma subgroups than microarrays. To systematically compare the power of RNA-seq and microarray-based models in predicting clinical endpoints, we divide the cohort randomly into training and validation sets and develop 360 predictive models on six clinical endpoints of varying predictability. Evaluation of factors potentially affecting model performances reveals that prediction accuracies are most strongly influenced by the nature of the clinical endpoint, whereas technological platforms (RNA-seq vs. microarrays), RNA-seq data analysis pipelines, and feature levels (gene vs. transcript vs. exon-junction level) do not significantly affect performances of the models.

**Conclusions:**

We demonstrate that RNA-seq outperforms microarrays in determining the transcriptomic characteristics of cancer, while RNA-seq and microarray-based models perform similarly in clinical endpoint prediction. Our findings may be valuable to guide future studies on the development of gene expression-based predictive models and their implementation in clinical practice.

**Electronic supplementary material:**

The online version of this article (doi:10.1186/s13059-015-0694-1) contains supplementary material, which is available to authorized users.

## Background

Microarray-based gene expression profiling is being widely applied in cancer research to identify biomarkers for clinical endpoint prediction, such as diagnosis, prognosis, or prediction of treatment response [1–5]. The clinical value of some of these classifiers is currently being examined in prospective trials [6]. Within the MicroArray Quality Control (MAQC)-II study [7], we observed, however, that the performance of gene expression models in predicting clinical outcome was limited and largely dependent on the respective clinical endpoint.

The advent of next-generation sequencing technologies has revolutionized eukaryotic transcriptome analysis. RNA deep-sequencing (RNA-seq) provides a powerful tool to decipher global gene expression patterns far beyond the limitations of microarrays, including an unprecedented capability to discover novel genes, alternative transcript variants, chimeric transcripts, and expressed sequence variants as well as allele-specific expression [8–12]. RNA-seq data have also been used to develop gene expression-based predictive models in cancer research [13, 14]. Considering the vast amount of additional information provided by RNA-seq in comparison to microarrays, it is tempting to speculate that RNA-seq-based models may outperform microarray-based models for clinical endpoint prediction. A comprehensive comparison of RNA-seq and microarray-based predictive models, however, is lacking to date.

In this study of the Sequencing Quality Control (SEQC) consortium, we therefore aimed to systematically investigate the potential of RNA-seq-based classifiers in predicting clinical endpoints in comparison to microarrays. To this end, we selected neuroblastoma as a model, a pediatric malignancy arising from the developing sympathetic nervous system [15]. The clinical courses of neuroblastoma are remarkably heterogeneous ranging from spontaneous regression to relentless progression. According to its diverse clinical presentations, patients are stratified into different prognostic subgroups, with therapeutic strategies ranging from ‘wait-and-see’ approaches to intensive multimodal treatments. Thus, accurate prediction of the natural course of the disease is an essential prerequisite for risk estimation and tailoring therapy intensities in individual patients. Treatment stratification in neuroblastoma is currently based on a combination of clinical and molecular parameters, including tumor stage, patient age at diagnosis, and the genomic amplification status of the *MYCN* proto-oncogene. In addition, a number of microarray-based gene expression models have been proposed to predict neuroblastoma patient outcome [16, 17]. However, while predictive models were highly accurate in risk assessment of current low- and intermediate-risk patients [18], the prediction of high-risk patient outcome has remained challenging [18–20].

Here, we determined global gene expression profiles from 498 primary neuroblastoma samples using both RNA-seq and Agilent’s 44 k oligonucleotide-microarrays to compare the performance of RNA-seq and microarray-based models in predicting clinical endpoints. We generated 360 gene expression-based models using a broad range of algorithms to predict six different endpoints with a varying degree of predictability, and analyzed the effects of a range of variables on the prediction performances. We found that prediction accuracies were most strongly influenced by the nature of the clinical endpoint, whereas neither the expression profiling technology nor the RNA-seq data analysis pipeline affected prediction accuracy systematically. To our knowledge, we present the first study on the evaluation of predictive models using RNA-seq in comparison to microarrays, which may provide valuable information for designing future experiments on gene expression-based classifiers using high-throughput technologies.

## Results

### Characterization of the neuroblastoma transcriptome using RNA-seq

To comprehensively characterize the neuroblastoma transcriptome, we sequenced 30,753,066,000 reads from 498 primary neuroblastoma samples covering the entire spectrum of the disease (Table 1). Discontinuous alignment of sequence reads to the genome revealed that 98.86 % of the reads mapped to the reference (Additional file 1: Figure S1; Additional file 2). We found that 348.5 Mbp (11.26 %) of the genome were expressed in neuroblastoma at a coverage threshold of 200, 197.7 Mbp (6.39 %) of which represented exonic regions (Additional file 1: FigureS2a). Within the expressed exome, 130.9 Mbp covered annotated genes (coding, 40.5 Mbp; non-coding, 90.3 Mbp), while 66.8 Mbp of the genome represented exonic regions not annotated in any of the databases RefSeq/EntrezGene [21], AceView [22], or Gencode [23] (coding regions, 6.8 Mbp; non-coding regions, 60.0 Mbp; Additional file 1: Figure S2b), corresponding to 39,052 novel exons supported by 118.4 Gbp (4.42 %) of the entire sequence information of our study (Additional file 1: Figure S2c).

**Table 1:** Clinical characteristics of neuroblastoma patients

	Number	Percent of total
*MYCN* status		
Normal	401	80.5 %
Amplified	92	18.5 %
N.A.	5	1.0 %
INSS stage		
1	121	24.3 %
2	78	15.7 %
3	63	12.7 %
4	183	36.7 %
4S	53	10.6 %
Age at diagnosis		
<18 months	300	60.2 %
>18 months	198	39.8 %
Sex		
Male	278	55.8 %
Female	205	41.2 %
N.A.	15	3.0 %
High-risk patients	176	35.3 %

NA, not available

**Fig. 1:**
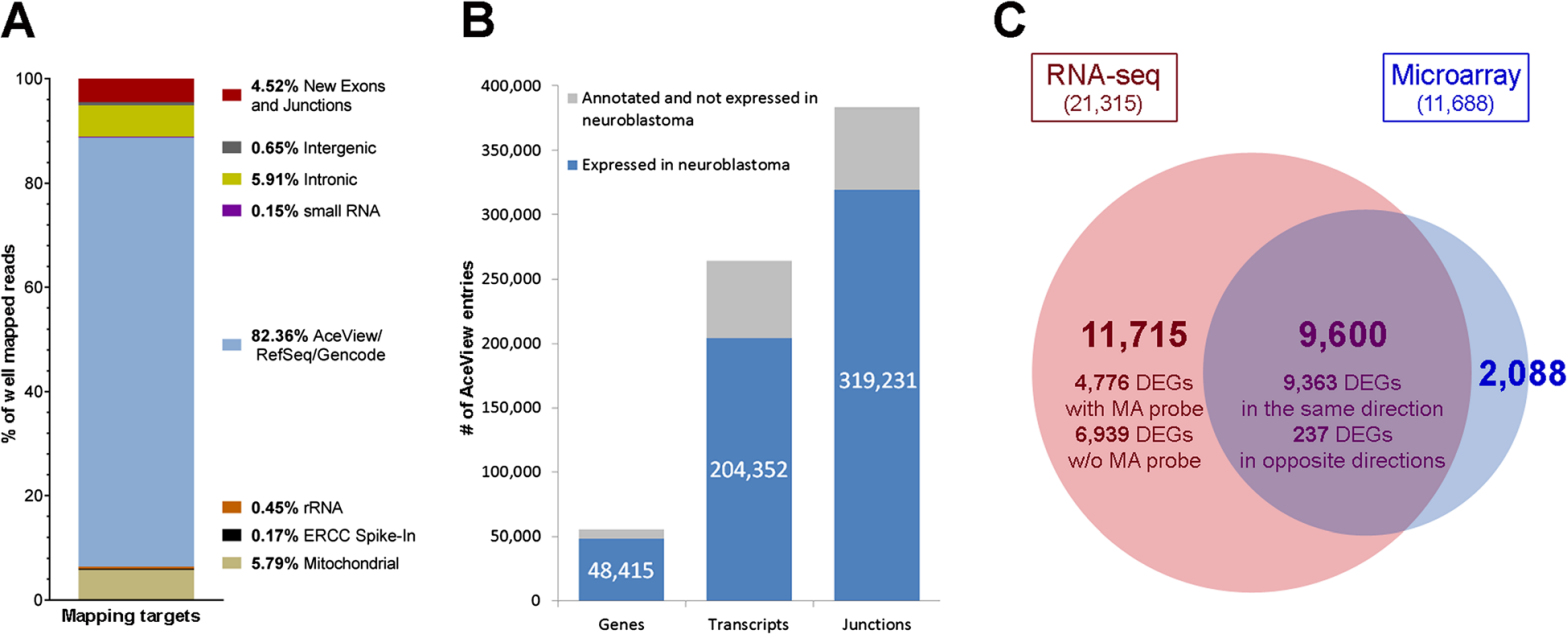
Characteristics of the neuroblastoma transcriptome according to RNA-seq data using the Magic-AceView pipeline. **a** Percentage of reads mapped to distinct targets. **b** Number of genes, transcripts, and exon-junctions expressed in the entire neuroblastoma cohort according to their annotation by AceView. **c** Absolute numbers and overlap of differentially expressed genes (DEGs) identified by RNA-seq (red) and microarrays (blue) in four disease subgroups (see main text)

In total, 88.75 % of the aligned reads mapped to genes annotated in either of the reference databases RefSeq (total number of genes annotated in the database, n = 24,536), AceView (n = 55,836), or Gencode (n = 56,071), while 4.52 % of the reads mapped to newly discovered exons or exon-junctions, 5.91 % to intronic and 0.65 % to intergenic sequences (Fig. 1a). In the entire neuroblastoma cohort, we identified 48,415 genes expressed above the background threshold when using AceView as a reference database (Fig. 1b), corresponding to an average of 28,490 (±1,399) expressed genes per sample (Additional file 1: Figure S3). By contrast, a total of 21,101 AceView genes were represented by the 44 k microarray used in this study. Among all genes detected in neuroblastoma, 21,933 represented genes encoding conserved proteins, 10,815 represented genes encoding mammalian-specific proteins, 1,427 genes were classified as marginally coding (that is, spliced genes with multiple alternative variants, of which only a minority appear to be protein-coding), and 14,240 represented non-coding genes. Furthermore, the mapped reads supported a total of 204,352 transcripts annotated in AceView, comprising 319,231 annotated exon-junctions (Fig. 1b; mapping results using the RefSeq and Gencode databases as references are given in Additional file 1: Figure S4).

### Analysis of differentially expressed genes (DEGs) in four neuroblastoma subgroups

Since gene expression-based prediction of clinical endpoints depends on differing mRNA levels in clinically relevant disease subgroups, we evaluated how analysis of RNA-seq data compares to microarrays in determining differential gene expression in four major clinico-genetic subtypes of neuroblastoma (Additional file 1: Table S1). We first restricted our analysis to the gene level considering only genes that were common to both platforms. DEGs were identified from both platforms using two different analytical methods: (1) a method based on the recommendations of the MAQC-I project utilizing a fold-change ranking and a non-stringent *P* value cutoff (MAQC-I) [7, 24, 25]; and (2) a novel method developed within the MAQC-III project utilizing the expression distributions, corrected for noise and batch effects, and assisted by random resampling, to compute DEG scores related to the Wilcoxon *U* test (Magic, see Additional file 1: Supplementary Note 2). On the platform level, we found that RNA-seq detected 5,488 (69.2 %) and 7,827 (67.0 %) of the DEGs identified by microarrays using the MAQC-I and Magic methods, respectively (Additional file 1: Figure S5). On the analytical method level, Magic detected 7,423 (93.5 %) and 6,728 (80.4 %) of the DEGs identified by the MAQC-I method using microarray and RNA-seq data, respectively (Additional file 1: Figure S5). Together, these results demonstrate that both the different platforms and the different analytical methods provide largely comparable results, indicating the validity of the methods used in our study.

To appreciate the comprehensive information provided by RNA-seq for differential gene expression analysis, we performed a second approach in which we applied the Magic pipeline on the full AceView-annotated transcript information in the four neuroblastoma subgroups. In total, we detected 54,164 differentially expressed transcripts corresponding to 21,315 DEGs using RNA-seq (RefSeq, n = 14,251; AceView only, n = 7,064; Additional file 3). By contrast, the 16,245 microarray probes found to be differentially represented in the four subgroups correspond to 11,688 DEGs (RefSeq, n = 10,308; AceView only, n = 1,380). Notably, RNA-seq analysis identified 80.1 % of the DEGs detected by microarrays (Fig. 1c), but in addition many more genes (annotated both in the RefSeq database and in the AceView database only) than microarrays (Additional file 1: Figure S6a). Furthermore, the power of RNA-seq in discovering tumor subtype-specific expression patterns became evident by detecting genes with discordant expression patterns, that is, genes with multiple transcript variants, of which at least one variant was differentially expressed while at least one other was not. We noted that 65.9 % of the 21,315 DEGs identified by RNA-seq showed such discordant expression patterns. As an example, we detected differentially expressed transcript variants of the cancer genes *NF1* and *MDM4* (Additional file 1: Figure S7 and S8). Both variants have been previously described to be of functional relevance in other cancer entities [26, 27], and were identified by the transcript-based approach only, while the gene-level analysis failed to report the two genes as DEGs. As a particular subgroup of genes with discordant expression patterns, we also determined DEGs of which at least one transcript variant was upregulated while at least one other variant was downregulated in the same subgroup. In this category, we identified 1,073 DEGs by RNA-seq, as opposed to 129 DEGs by microarrays (Additional file 1: Figure S6b). Focusing on cancer census genes [28], we observed such complex expression patterns for 26 of the current 513 cancer census genes (Additional file 1: Table S2). Together, our findings substantiate that RNA-seq is a powerful tool to determine the complex transcriptomic characteristics of cancer.

### Generation of predictive models from RNA-seq and microarray expression data

Given the comprehensive transcriptomic information provided by RNA-seq and its power to identify DEGs, we hypothesized that RNA-seq may improve gene expression-based clinical endpoint prediction over microarrays. To evaluate this hypothesis, we utilized RNA-seq and microarray-based expression data of all 498 primary neuroblastoma samples to predict six clinical endpoints: patients’ sex (SEX); the belonging to a patient subgroup with extreme disease outcome (referred to as CLASS LABEL, that is, event-free survivors without chemotherapy for at least 1,000 days post diagnosis [favorable], or patients died from disease despite chemotherapy [unfavorable]); the occurrence of events, that is, progression, relapse, or death (EFS ALL); the occurrence of death from disease (OS ALL); and the occurrence of events (EFS HR) and death from disease (OS HR) in the subset of current high-risk patients (that is, patients with stage 4 disease >18 months at diagnosis and patients of any age and stage with *MYCN*-amplified tumors; Table 2).

**Table 2:** Definition of clinical endpoints analyzed in this study

Cohort	Endpoint (bin 1/0)	Training set			Validation set		
		# Samples	1	0	# Samples	1	0
All patients (498)	SEX	249	103	146	249	108	141
	(Female/Male)						
	EFS ALL	249	89	160	249	94	155
	(Event yes/no)						
	OS ALL	249	51	198	249	54	195
	(Death yes/no)						
Class labeled patients (272)	CLASS LABEL	136	45	91	136	46	90
	(Unfavorable/Favorable)						
High-risk patients (176)	EFS HR	86	55	31	90	65	25
	(Event yes/no)						
	OS HR	86	43	43	90	49	41
	(Death yes/no)						

EFS, event-free survival; HR, high risk; OS, overall survival

Analogous to the strategy used in the MAQC-II study [7], the following best practice strategies were applied in model development and validation to obtain reliable and robust results: (1) Considering the fact that the nature of the clinical endpoint strongly affects a classifier’s performance [7, 18], we included endpoints that are known to cover a broad range of predictive difficulties (low difficulty, SEX, and CLASS LABEL; intermediate difficulty, EFS ALL, and OS ALL; high difficulty, EFS HR, and OS HR). (2) We involved six data analysis teams and applied various classification methods to take into account that the proficiency of data analysis teams and the choice of the classifier may impact the prediction results. (3) We implemented a two-step modeling strategy to ensure an unbiased validation: first, the models were developed based on a training set and frozen; then, the validation set was released for evaluation of the frozen models. (4) We decided to focus not only on RefSeq-annotated genes being the primary source of common microarrays designs, but also to consider more comprehensive transcriptomic information provided by the AceView database.

**Fig. 2:**
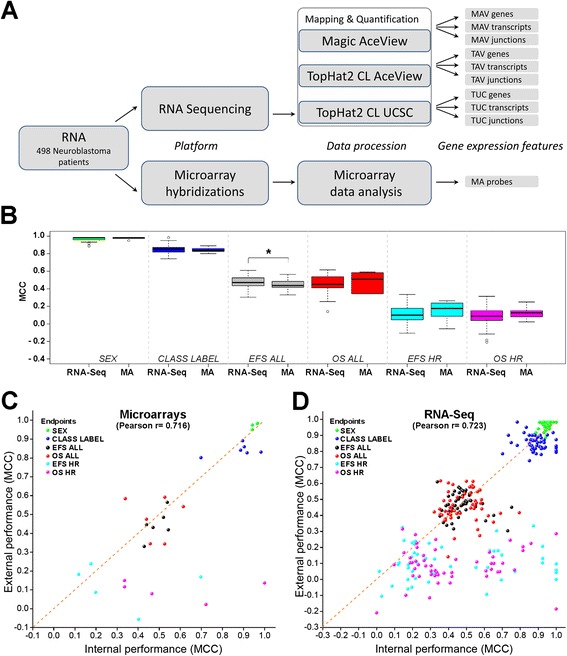
Performances of RNA-seq- and microarray-based models to predict clinical endpoints in the validation cohorts. **a** Schematic overview of gene expression profiles generated by RNA-seq (n = 9 per sample) and microarray (n = 1 per sample). CL, Cufflinks; MAV, Magic-AceView; TAV, TopHat-AceView; TUC, TopHat-UCSC. **b** Distribution of MCC values of all models for each endpoint according to the technical platform (MA, microarray). Boxes indicate the 25 % and 75 % percentiles, and whiskers indicate the 5 % and 95 % percentiles; (*), *P* <0.05; two-sided *T*-test was performed for statistical testing. **c**, **d** Model performance of internal validation compared with external validation based on (**c**) microarray and (**d**) RNA-seq expression data in terms of MCC

We extracted gene expression profiles from raw RNA-seq data using three different processing pipelines for mapping and quantifying sequence reads to also take the potential influence of RNA-seq data processing on prediction performances into account (Fig. 2a): (1) mapping reads to the AceView reference using the Magic alignment tool (MAV); (2) mapping reads to AceView using TopHat2 and Cufflinks (TAV); and (3) mapping reads to the UCSC database together with RefSeq gene annotations using TopHat2/Cufflinks (TUC). From the resulting data, we extracted gene expression profiles on three different feature levels: (1) gene, (2) transcript, and (3) exon-junction levels. Accordingly, each sample was associated with 10 different expression profiles, including one profile derived from microarray analyses (Fig. 2a). Neuroblastoma samples were randomly divided into training and validation sets (Table 2). Data analysis teams generated predictive models using their methods of choice, and submitted their best model for each of the six different endpoints on every expression profile, resulting in a total of 360 models. Afterwards, the models were used to predict endpoints in the validation set, and the external performance of each model was evaluated after unblinding the clinical information using various performance metrics (Additional files 4 and 5).

### Clinical endpoint prediction by RNA-seq- and microarray-based models

We determined prediction performances of all models in terms of MCC for each endpoint in the validation set, and compared performances of RNA-Seq and microarray-based models. RNA-seq-based models performed significantly better than microarray-based models in predicting endpoint EFS ALL (*P* = 0.043), while models based on the two platforms performed similarly well in predicting the remaining five endpoints (Fig. 2b).

In line with our findings from the MAQC II study [7], we noticed that prediction performances were strongly influenced by the respective clinical endpoint. While patient’s SEX and CLASS LABEL were predicted accurately, performances were substantially inferior for predicting EFS and OS in the entire cohort, and even worse in the high-risk subgroup by both RNA-seq and microarray-based models (Fig. 2b). To assess the potential clinical value of gene expression-based outcome prediction, we performed univariate Cox regression analysis and Kaplan-Meier survival estimates for patients of the validation set using the best performing RNA-seq and microarray models. The models significantly discriminated patients with favorable and unfavorable outcome in the entire cohort and in the class labeled cohort (Additional file 1: Figure S9a-d, Table S3 and S4), which is consistent with previous observations made by us and others [16–18, 20]. In the high-risk cohort, the best performing models were also able to predict patient outcome, which was in contrast to the prognostic markers age, stage and *MYCN* status (Additional file 1: Figure S9e and f, Fig. S10, and Table S5). The potential relevance of gene expression-based outcome prediction for neuroblastoma patients was substantiated by multivariate Cox regression analysis (Additional file 1: Table S6).

We also assessed whether performances observed in the validation set could have been estimated from performances determined in the internal cross-validation processes (Fig. 2c and d). Strikingly, correlations of internal and external validation performances for microarray- and RNA-seq-based models were almost identical (r = 0.716 and r = 0.723, respectively), indicating that the technical platform does not affect the reliability of performance estimates obtained during model training. It has to be noted, though, that performances of both RNA-seq- and microarray-based models for endpoints EFS HR and OS HR tended to be biased strongly towards internal validation, which is indicative of model overfitting on the training set. These results suggest that the endpoint itself influences the reliability of performance estimates derived from training cohorts.

### Variables affecting prediction performances of RNA-seq-based models

**Fig. 3:**
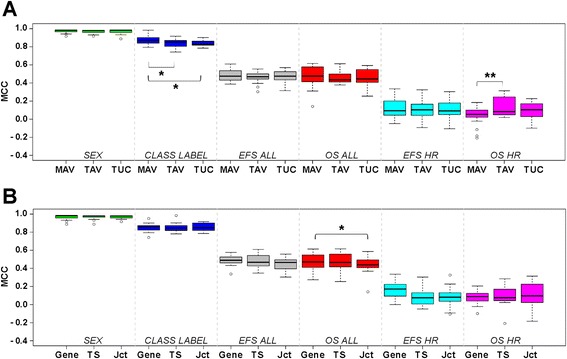
Analysis of factors potentially affecting prediction performances of RNA-seq-based models. **a** Distribution of MCC values of all models for each endpoint according to RNA-seq data processing pipelines (MAV, Magic-AceView; TAV, TopHat-AceView; TUC, TopHat-UCSC). **b** Distribution of MCC values of all models for each endpoint according to feature levels, that is, gene, transcript (TS), and exon-junction (Jct) levels

**Fig. 4:**
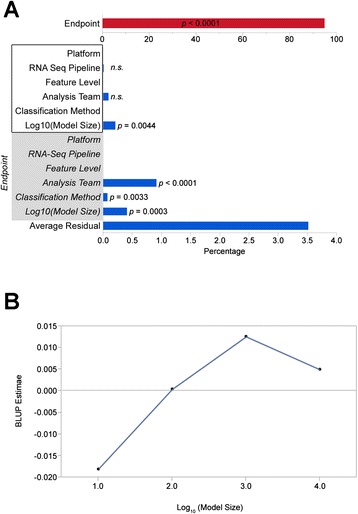
**a** Contribution of different factors to the variability of prediction results as assessed by variance component analysis. (*), *P* <0.05; (**), *P* <0.01. The factors platform, RNA-seq pipeline, feature level, analysis team, classification method, and model size were analyzed both independently of the endpoint (white box), and taking a potential endpoint-dependence into account (gray box). **b** Best linear unbiased predictor (BLUP) estimates for the log10(model size) as the single factor contributing significantly to the prediction variability independent of the endpoint. Note that BLUPs are centered around zero and effectively average over all other effects. BLUPs for Log10(Model Size) indicate that models with 100 to 1,000 features perform better than those with fewer or more features

We next aimed to identify additional variables that may affect prediction performances of RNA-seq-based models. In general, models derived from distinct processing pipelines and distinct feature levels did not differ significantly in their prediction performances with few exceptions (Fig. 3a and b). To investigate systematically which variables may affect model performances, we performed variance component analysis using Mixed Modeling [29]. We found that 95 % of the variation across MCC values was caused by effects of the endpoints, with lowest variances observed for endpoints predicted most accurately (Fig. 4a; Additional file 1: Table S7). Of the remaining 5 % of variability, 1.5 % was explained by four statistically significant effects. The size of the model (that is, the number of features included in the model) was the only factor that significantly affected prediction performances independent of the endpoint. On average, models comprising 100 to 1,000 features gave the best prediction results (Fig. 4b). The model size effect, however, differed between the endpoints analyzed: For prediction of the two endpoints that were easy to predict (SEX and CLASS LABEL), only one to 10 features were required, while more complex models improved prediction results for the remaining endpoints (Additional file 1: Figure S11a). In addition, different analysis teams and different modeling methods had varying performances across the endpoints (Additional file 1: Figure S11b and c). The remaining 3.5 % were residual variance not explained by any of the factors under investigation (Fig. 4a). Taken together, our data demonstrate that microarray and RNA-seq models perform similar in clinical endpoint prediction, and that the RNA-seq processing pipeline and the feature level do not influence prediction performances systematically.

**Fig. 5:**
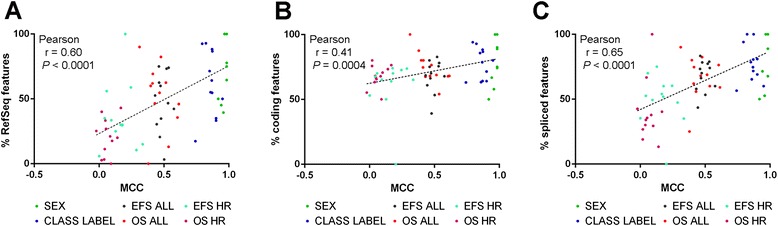
Correlation of prediction performances with the feature composition of prediction models. MCC values of MAV and TAV models were plotted against the fraction of RefSeq-annotated genes (**a**), the fraction of protein-coding genes (**b**), and the fraction of spliced genes (that is, genes or transcripts consisting of at least two exons; (**c**) in the model

We finally aimed to evaluate how different parts of the transcriptome contribute to the performance of predictive models. For this purpose, we determined the fraction of RefSeq-annotated features, protein coding features, and spliced features in MAV and TAV models both on the gene and transcript level. We found that the proportion of coding and spliced features in the models largely reflected the proportion of coding and spliced features in the AceView database (Additional file 1: Figure S12 and Table S8). Similarly, the proportion of RefSeq-annotated features in the models was in the range of their proportion in the AceView database. Taking all endpoints into account, however, we observed that prediction accuracies were significantly correlated with the fraction of RefSeq features, coding features, and spliced features in the models both on the gene and the transcript level (Fig. 5, Additional file 1: Figure S13). These data indicate that the composition of prediction models depends on the predictability of the clinical endpoints in general.

## Discussion

Here, we evaluated the potential of RNA-seq to predict clinical endpoints in comparison to microarrays. We generated gene expression profiles from 498 primary neuroblastoma samples using RNA-seq and microarrays, which represents, to the best of our knowledge, the most comprehensive description of a single cancer entity’s transcriptome. We demonstrate that gene expression profiles of neuroblastoma are tremendously complex, corresponding to findings on the transcriptomic landscape of other human cells published recently [9, 12, 30]. In the entire neuroblastoma cohort, we found 48,415 genes and 204,352 transcripts to be expressed, comprising 86.7 % and 77.3 % of all features annotated in the AceView database, respectively. We also identified >39,000 novel exons to be expressed in neuroblastoma, providing further evidence that the human transcriptome still exceeds the complexity reflected by current reference databases such as RefSeq, Gencode, and AceView. The comparison of gene expression profiles of four major clinico-genetic subgroups revealed that RNA-seq identified almost twice as many DEGs as microarrays. Of note, DEGs determined by RNA-seq comprised 80.1 % of the DEGs detected by microarrays, pointing towards the reliability of identifying DEGs by either method. One reason for the discrepant numbers received by RNA-seq and microarrays derives from the fact that 6,939 DEGs identified by RNA-seq were not represented by a probe on the microarray. In addition, 4,776 DEGs were not detected by microarrays although the genes were represented by a probe, which may be at least partly attributed to our analytical approach which was taking expression profiles at the transcript level into account. Taken together, our study substantiates that RNA-seq is capable of providing much more detailed insights into the transcriptomic characteristics of neuroblastoma than microarrays.

To systematically compare the potential of RNA-seq- and microarray-based models for clinical endpoint prediction, we utilized various data annotation pipelines and considered different feature levels to establish nine expression profiles per sample derived from RNA-seq data, complemented by one expression profile derived from microarray analyses. We generated 360 predictive models for six endpoints covering a broad range of prediction difficulties. Evaluation of the prediction performances in the validation set revealed that the endpoint represents the most relevant factor affecting model performances, which is well in line with the findings of the MAQC-II study [7]. By contrast, neither the technical platform (that is, RNA-seq vs. microarrays) nor the RNA-seq data annotation pipeline significantly affected the variability of prediction performances. Collectively, our data demonstrate that RNA-seq and microarray-based models perform similarly in clinical endpoint prediction.

We also noticed that models based on different feature levels predicted clinical endpoints with comparable accuracies. In turn, this result implies that models based on exon-junction levels perform equally well as models based on gene levels. These findings may impact the development of expression-based classifiers to be used in clinical settings, which are frequently transferred from high-throughput analyses to RT-qPCR-based assays [6, 20]: While assays based on gene expression levels may lack specificity due to uncertainties on the underlying relevant transcript variants, exon-junctions identified by RNA-seq provide an unambiguous source of expression information for developing specific diagnostic tests.

Our results do not support the hypothesis that the more extensive transcriptomic information provided by RNA-seq in comparison to microarrays may improve gene expression-based prediction performances in general. A possible explanation for this finding might be that the inherent complexity of RNA-seq data may promote over-fitting effects in the model development process, leading to over-optimistic internal prediction performances that cannot be reproduced in external validation cohorts [31]. We noted, however, that the correlation of internal and external validation performances was almost identical for RNA-seq and microarray-based models, indicating that over-fitting effects are independent of the technological platform. An alternative explanation for our results may be inferred from the observation that the proportion of RefSeq-annotated features in the prediction models was in the range of, or even above their proportion in the AceView database for most endpoints. This finding may suggest that the predictive information of RefSeq-annotated genes represented by standard microarrays is saturated, and that predictive information of more complex transcriptomic data provided by RNA-seq is largely redundant. It has to be noted, though, that models for endpoints that were difficult to predict (that is, EFS HR, OS HR) tended to disproportionately recruit features that are not annotated in RefSeq, suggesting that these features may considerably contribute to the prediction accuracy in these endpoints.

Both gene expression-based models derived from RNA-seq and microarray analyses were capable of predicting patient outcome in the entire neuroblastoma cohort accurately, thereby validating results from previous studies and underscoring their potential clinical utility for risk estimation in neuroblastoma [16–18, 20]. Notably, we observed that models containing 100 to 1,000 features on average performed better than models containing fewer features. This finding may argue against ambitious efforts to minimize feature numbers in predictive models, as has been done in the past [20, 32]. In addition, we found that the best performing models were able to predict outcome of high-risk patients with a similar precision as previously published multigene signatures [18, 20, 33], and independently from current prognostic markers. While the prognostic value of such multigene signatures needs to be validated in independent high-risk neuroblastoma cohorts, these findings may represent a starting point to establish biomarker-based risk assessment in this challenging patient subgroup.

## Conclusions

Our study demonstrates that RNA-seq based models are suitable for clinical endpoint prediction, and that prediction performances are similar to those of microarray-based models. Our findings may be used to guide the design of future studies for developing gene expression-based predictive models as well as their implementation in clinical practice. The key advantage of RNA deep-sequencing, however, resides in its ability to characterize transcriptomes at an unprecedented level of detail, which may lead to new insights into the molecular mechanisms of disease, thereby providing starting points for the development of rational targeted therapeutic strategies.

## Methods

### Patient samples

This project comprised tumor samples of 498 neuroblastoma patients from seven countries: Belgium (n = 1), Germany (n = 420), Israel (n = 11), Italy (n = 5), Spain (n = 14), United Kingdom (n = 5), and United States (n = 42). All patients were registered in respective clinical trials with informed consent. The patients’ age at diagnosis varied from 0 to 295.5 months (median age, 14.6 months). Tumor stage was classified according to the International Neuroblastoma Staging System (INSS): stage 1 (n = 121; *MYCN*-amplified (MNA), n = 3), stage 2 (n = 78; MNA, n = 5), stage 3 (n = 63; MNA, n = 15), stage 4 (n = 183; MNA, n = 65), stage 4S (n = 53; MNA, n = 4). Events were defined according to a revised version of the International Neuroblastoma Response Criteria [34].

### Gene expression analysis using oligonucleotide microarrays and RNA-sequencing

Tumor material preparation was performed as described previously [16]. For microarray analysis, gene expression profiles were generated using customized 4x44k oligonucleotide microarrays (Agilent Technologies). Sample preparation, labeling, and hybridization were performed according to the manufacturer’s protocol. Microarray expression profiles were generated using Agilent’s Feature Extraction software (Version 9.5.1) [35]. For RNA sequencing, Dynabeads® mRNA Purification Kit (Invitrogen) was used to purify mRNA from total RNA, and ERCC RNA spike-in was added according to the user guide. Library construction was performed according to the non-stranded TruSeqs™ protocol. Clusters were generated according to the TruSeq PE cluster Kit v3 reagent preparation guide (for cBot-HiSeq/HiScanSQ). High-throughput shotgun sequencing was performed on the Illumina HiSeq 2000 platform. Paired-end reads with lengths of 90 and 100 nucleotides were generated for 12 samples and 486 samples, respectively.

### Raw data preprocessing, read mapping, and gene expression quantification

In addition to the microarray expression profiles, three different RNA-seq processing pipelines were applied to generate expression profiles from the sequencing raw data.

1. The Magic-AceView pipeline (MAV) is based on the Magic analysis tool [22] (ftp://ftp.ncbi.nlm.nih.gov/repository/acedb/Software/Magic). Magic includes quality control, alignment, annotation, quantification, and normalization of RNA-seq data. Magic maps the read pairs in parallel on all targets, including the genome (NCBI 37), the RefSeq 37.104, the Gencode v15, and the AceView 2011 gene models. Gene expression is measured in sFPKM, adding to the standard FPKM (fragments per kilobase of transcript per million mapped reads) a gene-specific and experiment-specific threshold of significance (sFPKM) for low counts, together with a number of batch effect corrections. The Magic-AceView pipeline is described in detail as a Supplementary Note 1 (Additional file 1).
2. For the TopHat-AceView (TAV) pipeline, raw reads of RNA-seq were filtered using an in-house pipeline (BGI, Shenzhen, PR China). Clean RNA reads were aligned to the human genome (hg19) using TopHat [36]. Cufflinks was used to quantify the gene as well as transcript expression [37]. FPKM values for each annotated gene in the AceView database were calculated.
3. For the TopHat-UCSC pipeline (TUC), RNA-seq reads were mapped by TopHat [36] to a reference sequence consisting of the UCSC hg19 human genome and the RefSeq annotated genes. The mapped reads were processed by Cufflinks 1.3.0 with default parameters to assemble transcripts relying on RefSeq annotation [37]. Gene- and transcript-level expression values were computed by Cufflinks in terms of FPKM and transformed as log2(1 + FPKM) for downstream processing. Reads that align to known junctions were quantified by the Open Source software bam2ssj [38].

### Construction of classification models

Six data analysis teams received 10 expression profiles (nine profiles based on RNA-seq data and one profile based on microarray data) to predict six different endpoints as extensively described in the Results. Data analysts were asked to include a five-fold cross validation for 10 iterations to assess model performances in the training datasets, but were otherwise free in their choice of modeling algorithms and statistical tests to generate and select suitable prediction models. Models with highest average performance metrics were selected and submitted for testing on adequate blinded validation sets for each endpoint. More details and an overview of the applied classification algorithms are given in Additional file 1 (Supplementary Note 3 and Table S9).

### Prediction performance evaluation and statistical analyses

Matthew’s correlation coefficient (MCC) was used to evaluate the prediction performance of classification models as described [7]. Differences in model performances were subjected to variance component analysis using Mixed Modeling [29]. All the six major effects were assigned to random effects for partitioning their corresponding variances from total variance. Statistical F- and T- tests were applied for evaluating the significance of corresponding variance components and comparisons among the levels within each variance component, respectively. For statistical analysis of clinical data, IBM SPSS package release 20.0.0 and version 2.15.0 of the survival package in R was applied [39]. Overall survival (OS) was calculated as the time from diagnosis to death from disease or the last follow-up if the patient survived. Event-free survival (EFS) was calculated from diagnosis to the time of tumor progression, relapse, or death from disease or to the last follow-up if no event occurred. Survival curves were computed according to Kaplan-Meier estimates and compared with the log-rank test. Univariate Cox proportional hazards or logistic regression was applied with respect to EFS and OS to analyze the separate prognostic value of gene expression-based classification models or clinical markers, considering *P* <0.05 as significant. All analyses are regarded as explorative.

### Data availability

Microarray and RNA-seq data can be accessed from the GEO database (www.ncbi.nlm.nih.gov/geo/) with accession numbers GSE49710 and GSE49711, respectively, which are included in SEQC Project SuperSeries GSE47792.

## Additional files

Additional file 1:
**This file contains Supplementary Figures S1–S13, Supplementary Tables S1–S9, and Supplementary Notes 1–3 on the Magic-AceView pipeline, differential gene expression methods, and methodology on the generation of prediction models.**
Additional file 2:
**This file contains Supplementary Data: RNA-seq information of 498 neuroblastoma samples: Read fate and mapping targets.**
Additional file 3:
**This file contains Supplementary Data: Genes and transcript differentially expressed between four clinico-genetic neuroblastoma subgroups.**
Additional file 4:
**This file contains Supplementary Data: Internal cross-validation performance and external validation performance of 360 submitted prediction models.**
Additional file 5:
**This file contains Supplementary Data: Clinical characteristics and sample statistics for 498 neuroblastoma patients.**

## References

[1] Beer DG, Kardia SL, Huang CC, Giordano TJ, Levin AM, Misek DE (2002). Gene-expression profiles predict survival of patients with lung adenocarcinoma. Nat Med..

[2] Glas AM, Kersten MJ, Delahaye LJ, Witteveen AT, Kibbelaar RE, Velds A (2005). Gene expression profiling in follicular lymphoma to assess clinical aggressiveness and to guide the choice of treatment. Blood..

[3] Glinsky GV, Glinskii AB, Stephenson AJ, Hoffman RM, Gerald WL (2004). Gene expression profiling predicts clinical outcome of prostate cancer. J Clin Invest..

[4] Pomeroy SL, Tamayo P, Gaasenbeek M, Sturla LM, Angelo M, McLaughlin ME (2002). Prediction of central nervous system embryonal tumour outcome based on gene expression. Nature..

[5] Van’t Veer LJ, Dai H, Van de Vijver MJ, He YD, Hart AA, Mao M (2002). Gene expression profiling predicts clinical outcome of breast cancer. Nature..

[6] Reis-Filho JS, Pusztai L (2011). Gene expression profiling in breast cancer: classification, prognostication, and prediction. Lancet..

[7] Shi L, Campbell G, Jones WD, Campagne F, Wen Z, Walker SJ (2010). The MicroArray Quality Control (MAQC)-II study of common practices for the development and validation of microarray-based predictive models. Nat Biotechnol..

[8] Su Z, Łabaj PP, Li S, Thierry-Mieg J, Thierry-Mieg D, Shi W (2014). A comprehensive assessment of RNA-seq accuracy, reproducibility and information content by the Sequencing Quality Control Consortium. Nat Biotech..

[9] Djebali S, Davis CA, Merkel A, Dobin A, Lassmann T, Mortazavi A (2012). Landscape of transcription in human cells. Nature..

[10] Ozsolak F, Milos PM (2011). RNA sequencing: advances, challenges and opportunities. Nat Rev Genet..

[11] Sultan M, Schulz MH, Richard H, Magen A, Klingenhoff A, Scherf M (2008). A global view of gene activity and alternative splicing by deep sequencing of the human transcriptome. Science..

[12] Ferreira PG, Jares P, Rico D, Gomez-Lopez G, Martinez-Trillos A, Villamor N (2014). Transcriptome characterization by RNA sequencing identifies a major molecular and clinical subdivision in chronic lymphocytic leukemia. Genome Res..

[13] Cancer Genome Atlas Research Network (2013). Comprehensive molecular characterization of clear cell renal cell carcinoma. Nature.

[14] Volinia S, Croce CM (2013). Prognostic microRNA/mRNA signature from the integrated analysis of patients with invasive breast cancer. Proc Natl Acad Sci U S A..

[15] Maris JM, Hogarty MD, Bagatell R, Cohn SL (2007). Neuroblastoma. Lancet..

[16] Oberthuer A, Berthold F, Warnat P, Hero B, Kahlert Y, Spitz R (2006). Customized oligonucleotide microarray gene expression-based classification of neuroblastoma patients outperforms current clinical risk stratification. J Clin Oncol..

[17] Ohira M, Oba S, Nakamura Y, Isogai E, Kaneko S, Nakagawa A (2005). Expression profiling using a tumor-specific cDNA microarray predicts the prognosis of intermediate risk neuroblastomas. Cancer Cell..

[18] Oberthuer A, Hero B, Berthold F, Juraeva D, Faldum A, Kahlert Y (2010). Prognostic impact of gene expression-based classification for neuroblastoma. J Clin Oncol..

[19] Asgharzadeh S, Pique-Regi R, Sposto R, Wang H, Yang Y, Shimada H (2006). Prognostic significance of gene expression profiles of metastatic neuroblastomas lacking MYCN gene amplification. J Natl Cancer Inst..

[20] Vermeulen J, De Preter K, Naranjo A, Vercruysse L, Van Roy N, Hellemans J (2009). Predicting outcomes for children with neuroblastoma using a multigene-expression signature: a retrospective SIOPEN/COG/GPOH study. Lancet Oncol..

[21] Pruitt KD, Brown GR, Hiatt SM, Thibaud-Nissen F, Astashyn A, Ermolaeva O (2014). RefSeq: an update on mammalian reference sequences. Nucleic Acids Res..

[22] Thierry-Mieg D, Thierry-Mieg J (2006). AceView: a comprehensive cDNA-supported gene and transcripts annotation. Genome Biol..

[23] Harrow J, Frankish A, Gonzalez JM, Tapanari E, Diekhans M, Kokocinski F (2012). GENCODE: the reference human genome annotation for The ENCODE Project. Genome Res..

[24] Guo L, Lobenhofer EK, Wang C, Shippy R, Harris SC, Zhang L (2006). Rat toxicogenomic study reveals analytical consistency across microarray platforms. Nat Biotechnol..

[25] Shi L, Reid LH, Jones WD, Shippy R, Warrington JA, Baker SC (2006). The MicroArray Quality Control (MAQC) project shows inter- and intraplatform reproducibility of gene expression measurements. Nat Biotechnol..

[26] Lenos K, Grawenda AM, Lodder K, Kuijjer ML, Teunisse AF, Repapi E (2012). Alternate splicing of the p53 inhibitor HDMX offers a superior prognostic biomarker than p53 mutation in human cancer. Cancer Res..

[27] Nishi T, Lee PS, Oka K, Levin VA, Tanase S, Morino Y (1991). Differential expression of two types of the neurofibromatosis type 1 (NF1) gene transcripts related to neuronal differentiation. Oncogene..

[28] Futreal PA, Coin L, Marshall M, Down T, Hubbard T, Wooster R (2004). A census of human cancer genes. Nat Rev Cancer..

[29] Littell RC, Milliken GA, Stroup WW, Wolfinger RD, Schabenberger O (2006). SAS system for mixed models.

[30] Prensner JR, Iyer MK, Balbin OA, Dhanasekaran SM, Cao Q, Brenner JC (2011). Transcriptome sequencing across a prostate cancer cohort identifies PCAT-1, an unannotated lincRNA implicated in disease progression. Nat Biotechnol..

[31] Simon R (2005). Roadmap for developing and validating therapeutically relevant genomic classifiers. J Clin Oncol..

[32] Garcia I, Mayol G, Rios J, Domenech G, Cheung NK, Oberthuer A (2012). A three-gene expression signature model for risk stratification of patients with neuroblastoma. Clin Cancer Res..

[33] Stricker TP (2014). Morales La Madrid A, Chlenski A, Guerrero L, Salwen HR, Gosiengfiao Y, et al. Validation of a prognostic multi-gene signature in high-risk neuroblastoma using the high throughput digital NanoString nCounter system. Mol Oncol.

[34] Brodeur GM, Pritchard J, Berthold F, Carlsen NL, Castel V, Castelberry RP (1993). Revisions of the international criteria for neuroblastoma diagnosis, staging, and response to treatment. J Clin Oncol..

[35] Oberthuer A, Juraeva D, Li L, Kahlert Y, Westermann F, Eils R (2010). Comparison of performance of one-color and two-color gene-expression analyses in predicting clinical endpoints of neuroblastoma patients. Pharmacogenomics J..

[36] Trapnell C, Pachter L, Salzberg SL (2009). TopHat: discovering splice junctions with RNA-Seq. Bioinformatics..

[37] Trapnell C, Williams BA, Pertea G, Mortazavi AM, Kwan G, van Baren MJ (2010). Transcript assembly and quantification by RNA-Seq reveals unannotated transcripts and isoform switching during cell differentiation. Nat Biotech..

[38] Pervouchine DD, Knowles DG, Guigo R (2013). Intron-centric estimation of alternative splicing from RNA-seq data. Bioinformatics..

[39] Therneau T. A Package for Survival Analysis in S. R package version 2.36-12. 2012. Available at: http://CRAN.R-project.org/package=survival.

