# Supplementary material for: Comparison of RNA-seq and microarray-based models for clinical endpoint prediction
Source: Genome Biol. 2015 Jun 25;16(1):133. doi: 10.1186/s13059-015-0694-1 (PMC4506430; doi:10.1186/s13059-015-0694-1)
Supplement: Additional file 1: — This file contains Supplementary Figures S1–S13, Supplementary Tables S1–S9, and Supplementary Notes 1–3 on the Magic-AceView pipeline, differential gene expression methods, and methodology on the generation of prediction models. [file 13059_2015_694_MOESM1_ESM.pdf]

ADDITIONAL FILE 1

Comparison of RNA-seq and microarray-based models for clinical endpoint prediction

The SEQC consortium

Wenqian Zhang[#], Ying Yu[#], Falk Hertwig[#], Jean Thierry-Mieg[#], Wenwei Zhang[#], Danielle Thierry-Mieg, Jian Wang, Cesare Furlanello, Viswanath Devanarayan, Jie Cheng, Youping Deng, Barbara Hero, Huixiao Hong, Meiwen Jia, Li Li, Simon M Lin, Yuri Nikolsky, André Oberthuer, Tao Qing, Zhenqiang Su, Ruth Volland, Charles Wang, May D Wang, Junmei Ai, Davide Albanese, Shahab Asgharzadeh, Smadar Avigad, Wenjun Bao, Marina Bessarabova, Murray H Brilliant, Benedikt Brors, Marco Chierici, Tzu-Ming Chu, Jibin Zhang, Richard G Grundy, Min Max He, Scott Hebbring, Howard L Kaufman, Samir Lababidi, Lee J Lancashire, Yan Li, Xin X Lu, Heng Luo, Xiwen Ma, Baitang Ning, Rosa Noguera, Martin Peifer, John H Phan, Frederik Roels, Carolina Roßwog, Susan Shao, Jie Shen, Jessica Theissen, Gian Paolo Tonini, Jo Vandesompele, Po-Yen Wu, Wenzhong Xiao, Joshua Xu, Weihong Xu, Jiekun Xuan, Yong Yang, Zhan Ye, Zirui Dong, Ke K Zhang, Ye Yin, Chen Zhao, Russell D Wolfinger, Tieliu Shi, Linda H Malkas, Frank Berthold, Jun Wang, Weida Tong, Leming Shi*, Zhiyu Peng*, Matthias Fischer*

[#]These authors contributed equally to this work.

*Correspondence to:

Leming Shi, lemingshi@fudan.edu.cn; Zhiyu Peng, pengzhiyu@genomics.org.cn; Matthias Fischer, matthias.fischer@uk-koeln.de

PART A – Supplementary Figures (pp. 4 – 16)

Figure S1 – *Mapping statistics of the entire RNA-seq data according to the Magic alignment tool.*

Figure S2 – *Genome coverage of the neuroblastoma transcriptome.*

Figure S3 – *Number of genes and transcripts called as reliably expressed for all 498 individual neuroblastoma samples.*

Figure S4 – *Number of genes, transcripts, and exon junctions called to be expressed in the entire neuroblastoma cohort according to the RefSeq and Gencode databases.*

Figure S5 – *Comparison of platforms and analysis tools for the detection of differentially expressed genes.*

Figure S6 – *Genes differentially expressed between either of four neuroblastoma subgroups as identified by RNA-seq and microarrays.*

Figure S7 – *Differential expression of transcript variants of MDM4.*

Figure S8 – *Differential expression of transcript variants of NF1.*

Figure S9 – *Kaplan-Meier survival estimates of neuroblastoma patients classified to be favorable or unfavorable according to the best-performing RNA-seq-based (blue) and microarray-based (red) prediction models.*

Figure S10 – *Kaplan-Meier survival estimates of high-risk neuroblastoma patients according to established prognostic markers.*

Figure S11 – *Best linear unbiased predictor (BLUP) estimates for 3 factors contributing significantly to the prediction variability in addition to the endpoint.*

Figure S12 – *Feature composition of prediction models that were based on the AceView database (MAV and TAV models).*

Figure S13 – *Correlation of prediction performances with the feature composition of prediction models.*

Part B – Supplementary Tables (pp. 17 – 30)

Table S1 – *Definition of 4 major clinico-genetic neuroblastoma subgroups*

Table S2 – *Cancer census genes with a complex differential transcript expression pattern*

Table S3 – *Univariate Cox regression analysis of models performing best in terms of MCC in the entire validation set (n=249; endpoints EFS ALL, OS ALL)*

Table S4 – *Univariate logistic regression analysis of models performing best in terms of MCC in the class-labeled validation set (n=136; endpoint CLASS LABEL)*

Table S5 – *Univariate Cox regression analysis of models performing best in terms of MCC in the high-risk validation set (n=90; endpoints EFS HR, OS HR)*

Table S6 – *Multivariate Cox regression analysis of the single microarray- and RNA-seq-based models which performed best for each endpoint in terms of MCC*

Table S7 – *Variance component analysis of model prediction performances*

Table S8 – *Correlation of prediction performances and the feature composition with regard to the fraction of RefSeq, protein coding, and spliced features in MAV and TAV models*

Table S9 – *Classification algorithms selected by data analysis teams*

PART C – Supplementary Notes

SUPPLEMENTARY NOTE 1: The Magic RNA-seq Analysis Pipeline *(pp 31 – 39)*

SN1.1 – *Mapping*

SN1.2 – *Quantification of RNA-seq expression data*

SN1.3 – *Analysis of the annotated transcriptome*

SN1.4 – *Discovery of novel introns and exons*

SUPPLEMENTARY NOTE 2: Comparative analysis of differential gene expression *(pp 40 - 46)*

SN2.1 – *QC of the microarray design: probe quality assessment and mapping by Magic*

SN2.2 – *Methodology for differential expression: MAQC-I standard approach*

SN2.3 – *Methodology for differential expression: Magic pipeline approach*

SUPPLEMENTARY NOTE 3: Generation of prediction models *(pp 47 – 49)*

PART A – Supplementary Figures

Figure S1

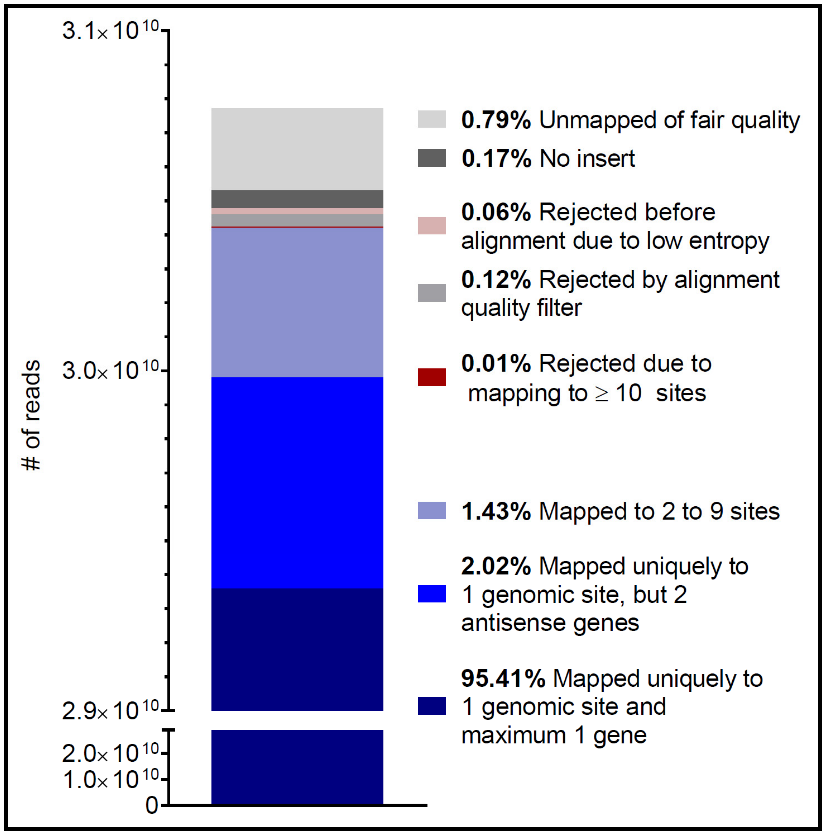

Mapping statistics of the entire RNA-seq data according to the Magic alignment tool.

Figure S2

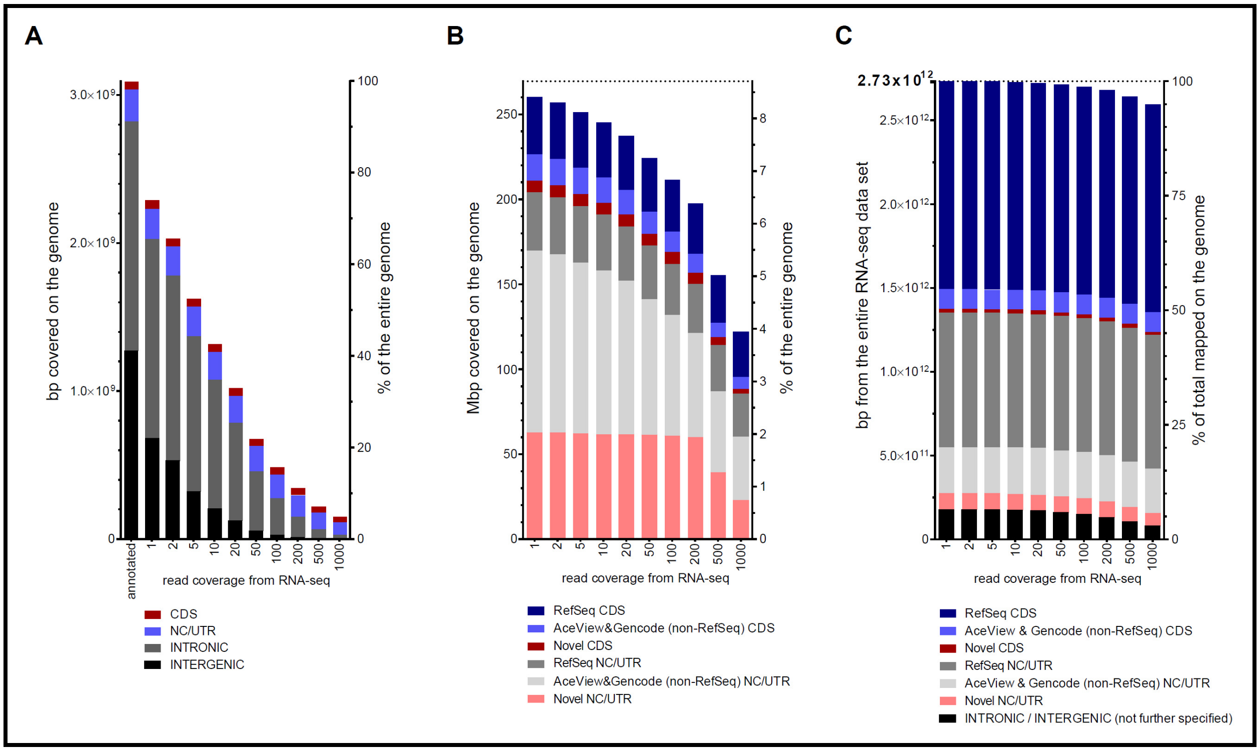

Genome coverage of the neuroblastoma transcriptome. Coverage of the human genome by neuroblastoma RNA-seq data on the basis of hierarchical mapping to coding > non-coding-or-UTR > intronic > intergenic then in each category to RefSeq > AceView or Gencode > genome [HG19]. (**A**) Proportion of coding regions, non-coding regions (including UTR of coding regions), intronic, and intergenic regions as a function of fold coverage. (**B**) Exonic coverage: Proportion of annotated coding regions and non-coding regions as well as newly discovered coding and non-coding regions. The dashed line indicates the entire annotated human transcriptome (all CDS + NC/UTR, 269.495,959 bp). (**C**) Coverage of the neuroblastoma transcriptome by RNA-seq data. As indicated by the dashed line (at 100%), $2.73x10^{12}$ bp of the ~3TB RNA-seq data were mapped to the genome. NC, non-coding; UTR, untranslated region; bp, base pairs. (See also Supplementary Note 1 [part C of this document] for the identification and definition of novel transcribed regions.)

Figure S3

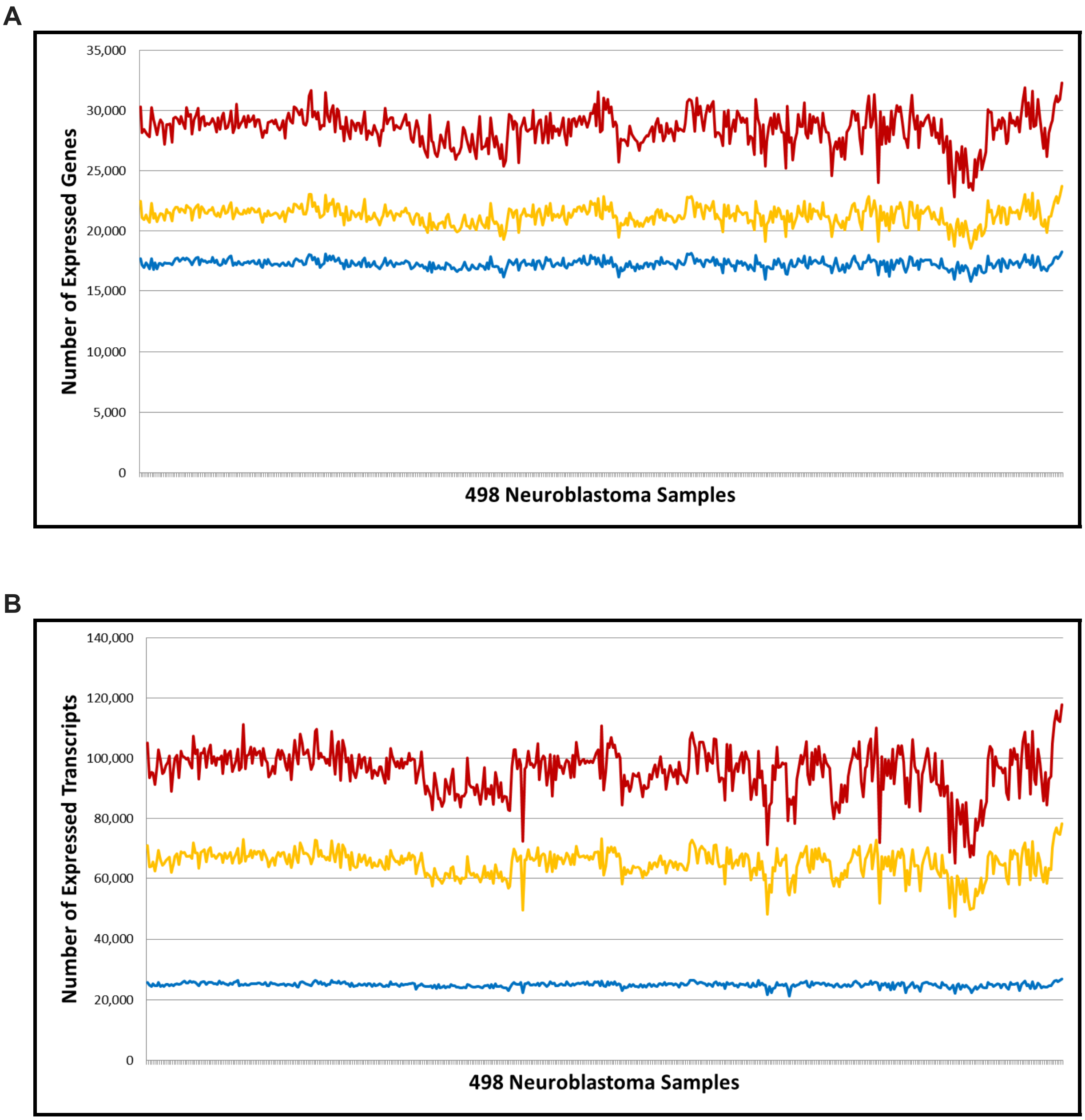

Number of genes (A) and transcripts (B) called as reliably expressed for all 498 individual neuroblastoma samples. The color code indicates the annotation: red, genes annotated in AceView (average, 28,491 genes and 95,824 transcripts per sample); blue, genes annotated in RefSeq (average, 17,274 genes and 24,911 transcripts per sample); yellow, genes annotated in Gencode (average, 21,286 genes and 65,093 transcripts per sample).

Figure S4

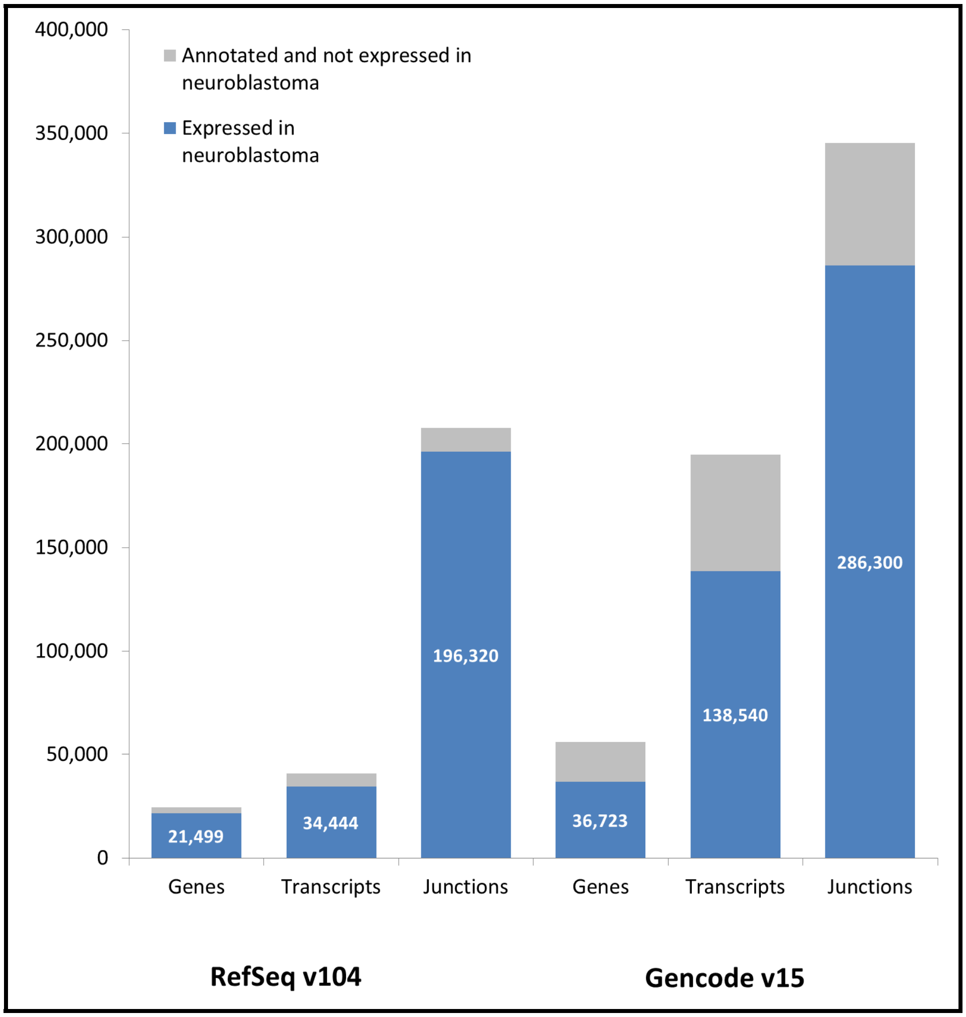

Number of genes, transcripts, and exon junctions called to be expressed in the entire neuroblastoma cohort according to the RefSeq (v104) and Gencode (v15) databases. Blue bars indicate genes, transcripts, and exon junctions expressed in neuroblastoma, while gray stacked bars indicate annotated genes, transcripts, or exon junctions for which no expression was detected in neuroblastoma.

Figure S5

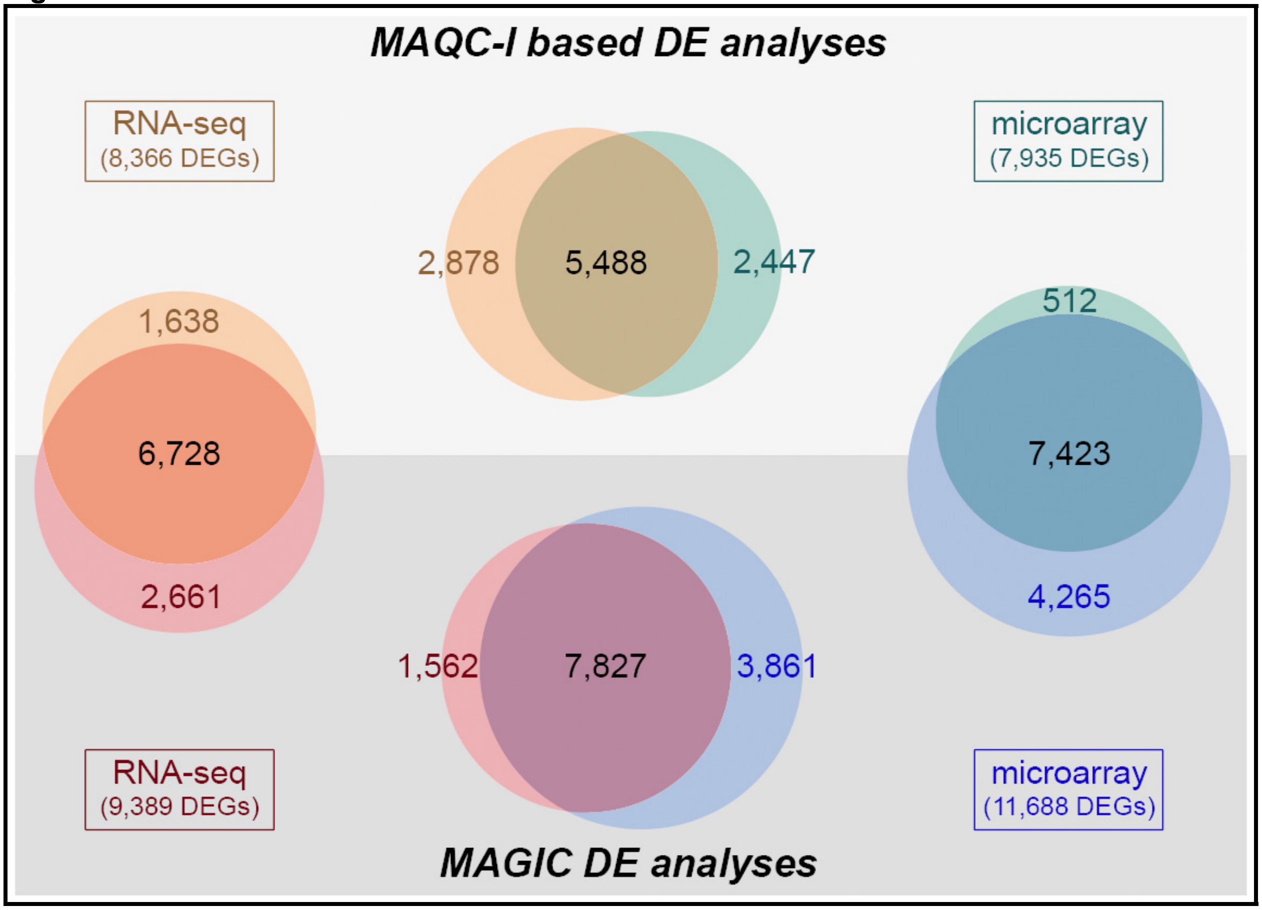

Comparison of platforms and analysis tools for the detection of differentially expressed genes (DEGs). Differential expression (DE) of four defined neuroblastoma subgroups (see also Table S1) was assessed from RNA-seq and microarray data at the gene level. Two DE pipelines (MAQC-I and Magic) were used (see also Supplementary Note 2). The numbers and the overlap of DEGs identified by each pipeline and platform are shown in Venn diagrams (MAQC-I approach: top, Magic approach, bottom; RNA-seq based DEGs, left side; DEGs identified by microarray analyses, right side). In comparison to the MAQC-I approach the Magic method identifies an increased number of DEGs overall, as well as an increased overlap of DEGs identified by both gene expression platforms. This comparative analysis is limited to the 21,101 genes assessed on Agilent microarrays by uniquely mapping probes.

Figure S6

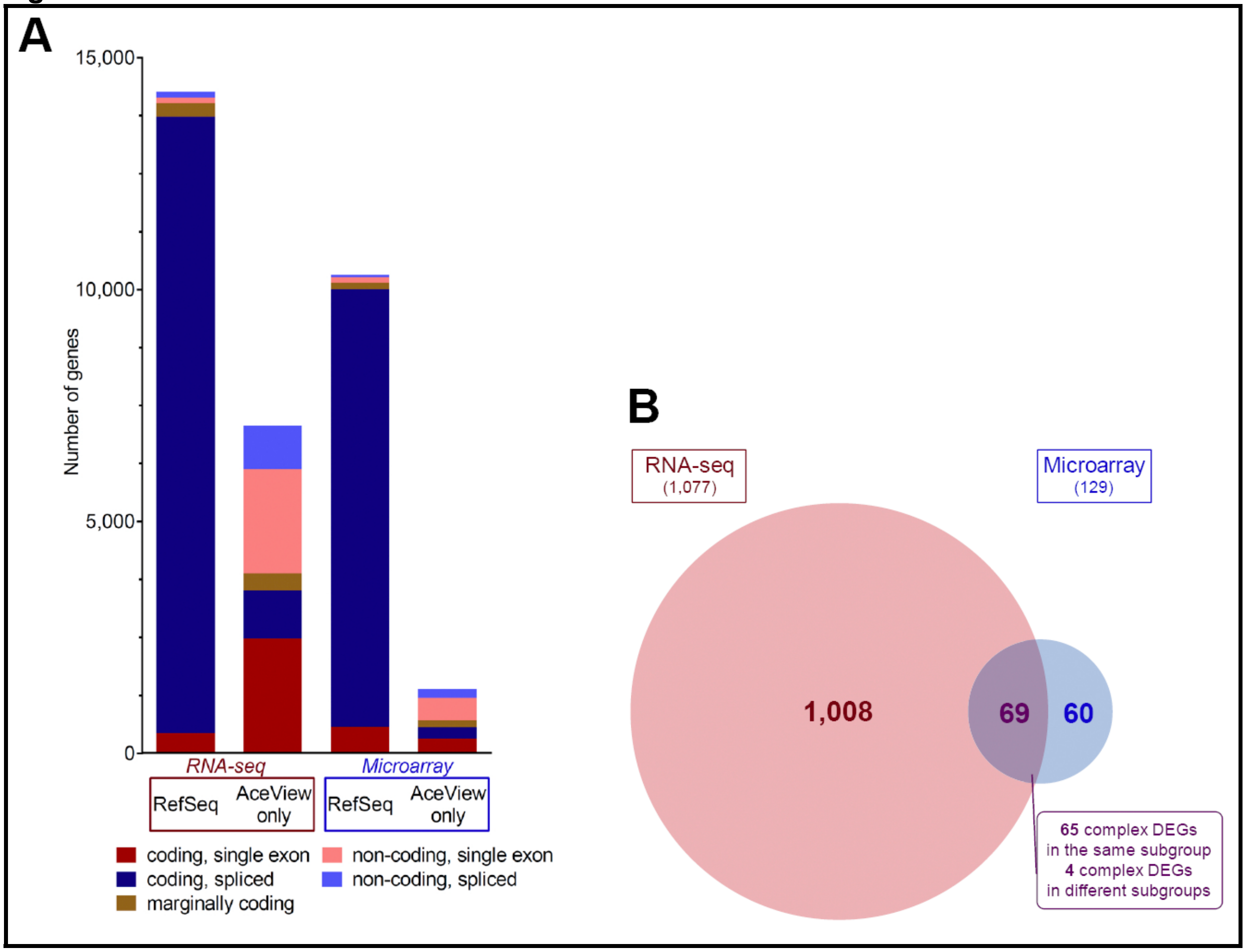

Genes differentially expressed (DEGs) between either of four neuroblastoma subgroups as identified by differential transcripts via RNA-seq and differential probes in microarrays. (**A**) DEGs identified by RNA-seq and by microarrays according to their annotation status (RefSeq vs. AceView-only), their structural complexity (single exon vs. multi-exon), and their protein-coding capacity. In this approach, DEGs were defined by the detection of differential expression (Magic method, Supplementary Note 2) on the single transcript and probe level by RNA-seq and microarrays, respectively (see also Fig. 1C). (**B**) Absolute numbers and overlap of complex regulated DEGs (i.e., different transcript variants of the same gene that are differentially expressed in opposite directions in the same clinical subgroup) identified by RNA-seq (red) and by microarrays (blue) in all subgroups.

Figure S7

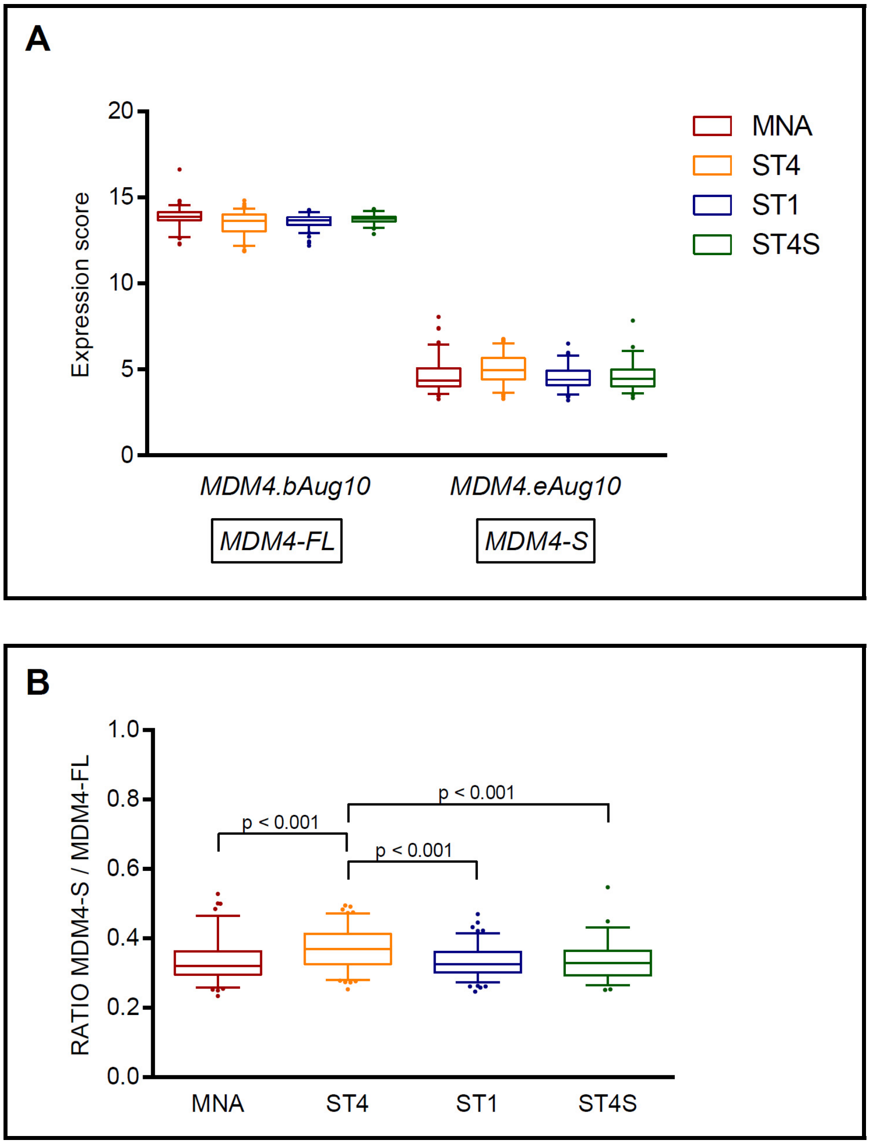

Differential expression of transcript variants of *MDM4*. (**A**) The type *MDM4*-S transcript (*MDM4.eAug10*) of the *MDM4* gene is up-regulated in stage 4 neuroblastomas. (**B**) The ratio of the transcript variants *MDM4-S*:*MDM4-FL* (*MDM4.bAug10*), which has previously been reported to correlate with increased proliferation, metastasis and poor prognosis in osteosarcoma and soft tissue sarcomas [1], is increased in stage 4 neuroblastoma.

Figure S8

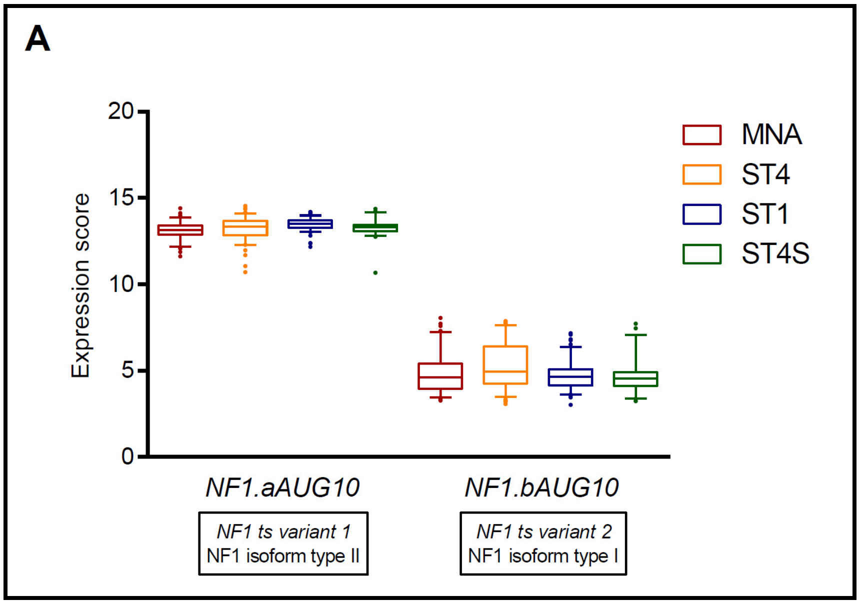

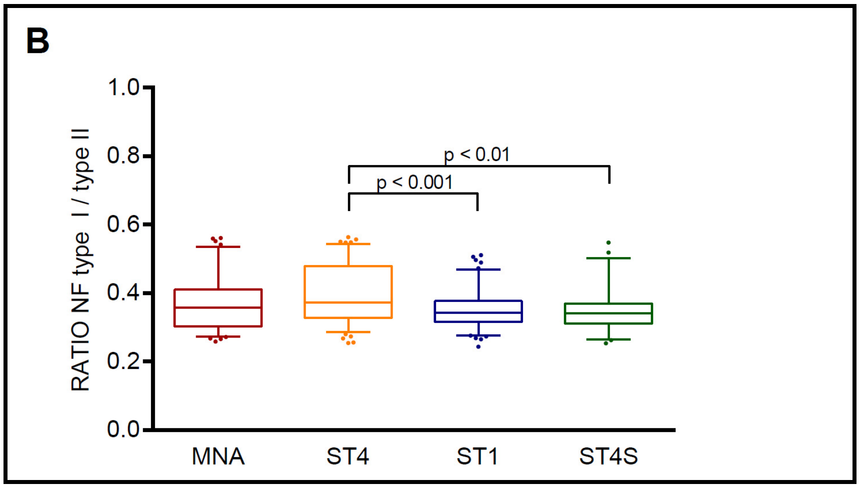

Differential expression of transcript variants of *NF1*. (**A**) The type I isoform (transcript variant 2: *NF1.bAug10*) of the neurofibromatosis 1 gene (*NF1*) is up-regulated in stage 4 neuroblastomas. (**B**) The ratio of NF1 type I : type II isoforms is increased in stage 4 neuroblastoma. Increased levels of type I isoforms and an increased ratio of type I : type II isoforms have been reported to be characteristic for immature neural tumor types [2].

Figure S9

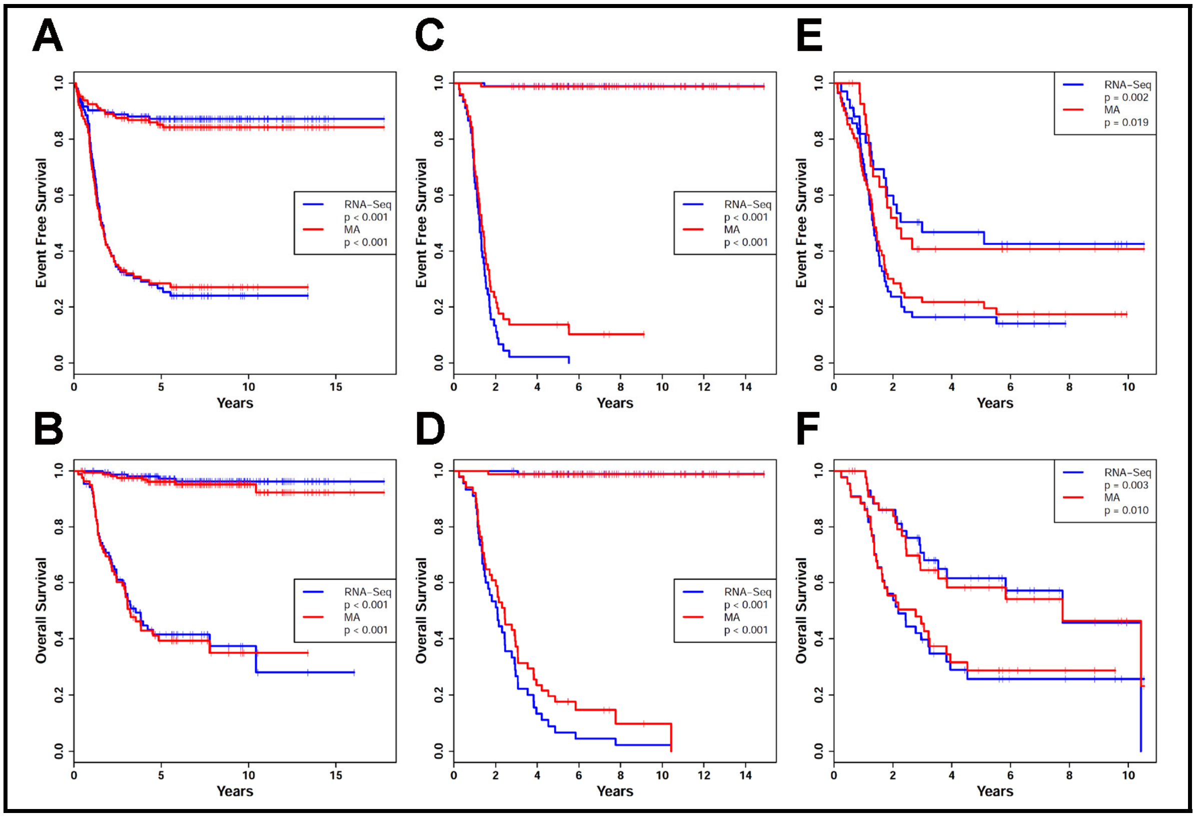

Kaplan-Meier survival estimates of neuroblastoma patients classified to be favorable or unfavorable according to the best-performing RNA-seq-based (blue) and microarray-based (red) prediction models. Survival curves according to (**A**) event-free survival (EFS) and (**B**) overall survival (OS) of patients of the entire validation set. Survival curves for (**C**) EFS and (**D**) OS of favorable/unfavorable class-labeled patients of the validation set. Survival curves for (**E**) EFS and (**F**) OS of high-risk patients of the validation set. RNA-seq and microarray models discriminated similarly well between patients with favorable *vs.* unfavorable outcome, as also indicated in the 5-year survival fractions (**A**, EFS at 5 years, 87.2±2.8 *vs.* 26.7±4.5 [RNA-seq] and 85.2±3.0 *vs.* 28.5±4.6 [microarrays]; **B**, OS at 5 years, 97.2±1.4 *vs.* 41.5±5.7 [RNA-seq] and 96.1±1.6 *vs.* 39.4±5.8 [microarrays], **C**, 5-year EFS, 98.9±1.1 *vs.* 2.2±2.2 [RNA-seq] and 98.8±1.2 *vs.* 13.7±4.8 [microarrays]; **D**, 5-year OS, 98.9±1.1 *vs.* 6.7±3.7 [RNA-seq] and 98.8±1.2 *vs.* 17.6±5.3 [microarrays]; **E**, 5-year EFS, 46.8±8.9 *vs.* 16.4±5.0 [RNA-seq] and 40.7±9.5 *vs.* 21.8±5.3 [microarrays]; **F**, 5-year OS, 61.6±8.0 *vs.* 25.7±7.0 [RNA-seq] and 58.2±7.9 *vs.* 28.7±7.3 [microarrays]).

Figure S10

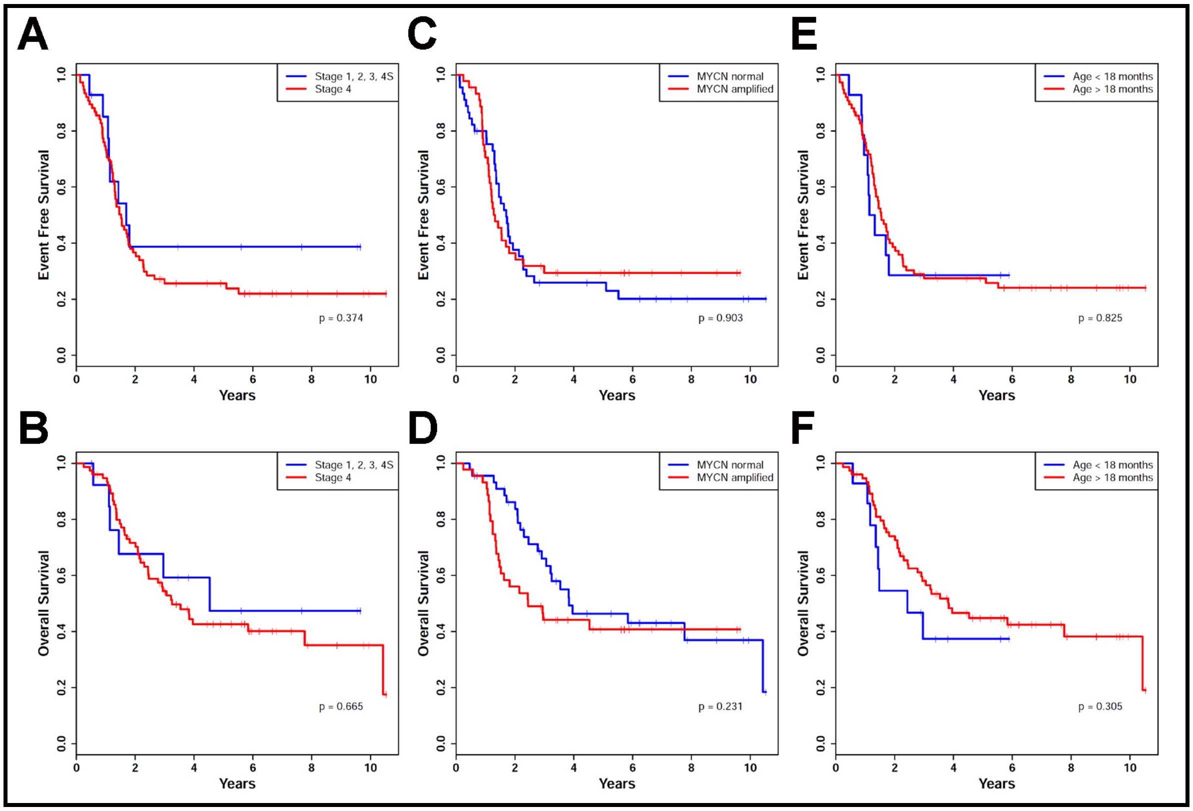

Kaplan-Meier survival estimates of high-risk neuroblastoma patients according to established prognostic markers. (**A, B**) Survival curves for (**A**) event-free survival (EFS) and (**B**) overall survival (OS) according to tumor stage (blue, stages 1, 2, 3, 4S; red, stage 4). (**C, D**) Survival curves for (**C**) EFS and (**D**) OS according to the *MYCN* amplification status (blue, tumors without *MYCN* amplification; red, *MYCN*-amplified tumors). (**E, F**) Survival curves for (**E**) EFS and (**F**) OS according to patients' age at diagnosis (blue, <18 months; red, >18 months).

Figure S11

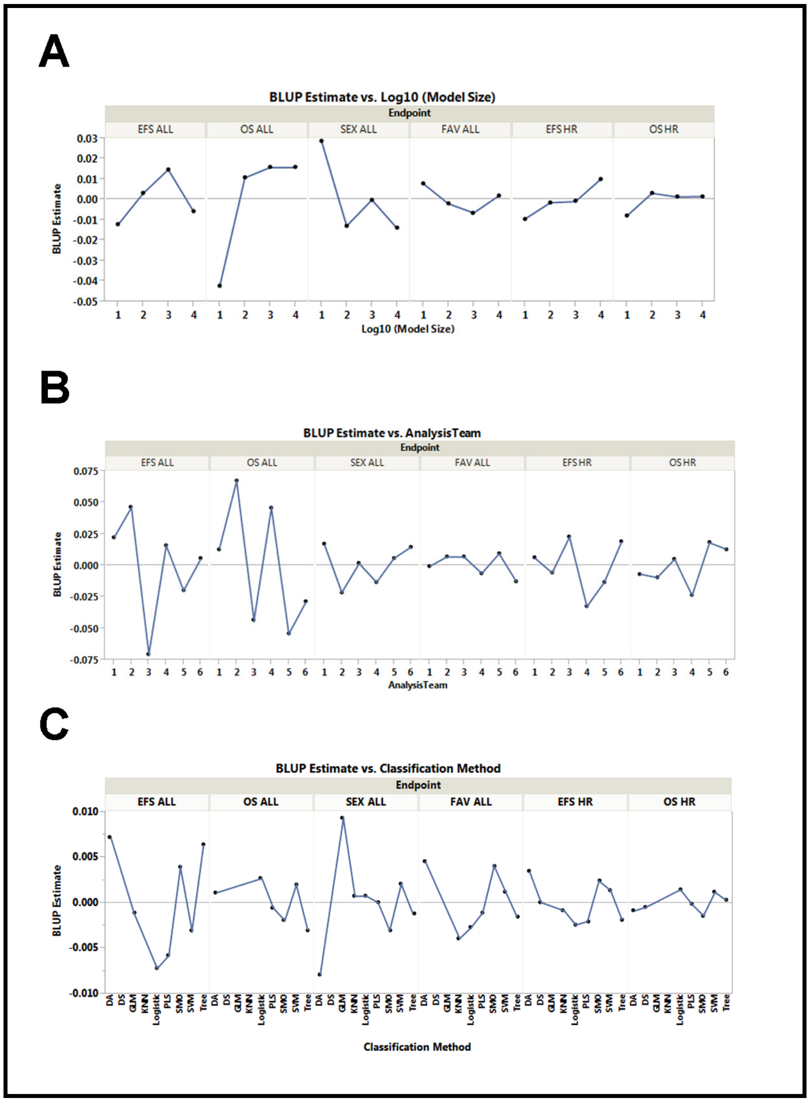

Best linear unbiased predictor (BLUP) estimates for 3 factors contributing significantly to the prediction variability in an endpoint-dependent manner. BLUP estimates for (**A**) endpoint*$\log_{10}$(model size), (**B**) endpoint*analysis team, and (**C**) endpoint*classification method. Model size refers to the number of features used in a prediction model.

Figure S12

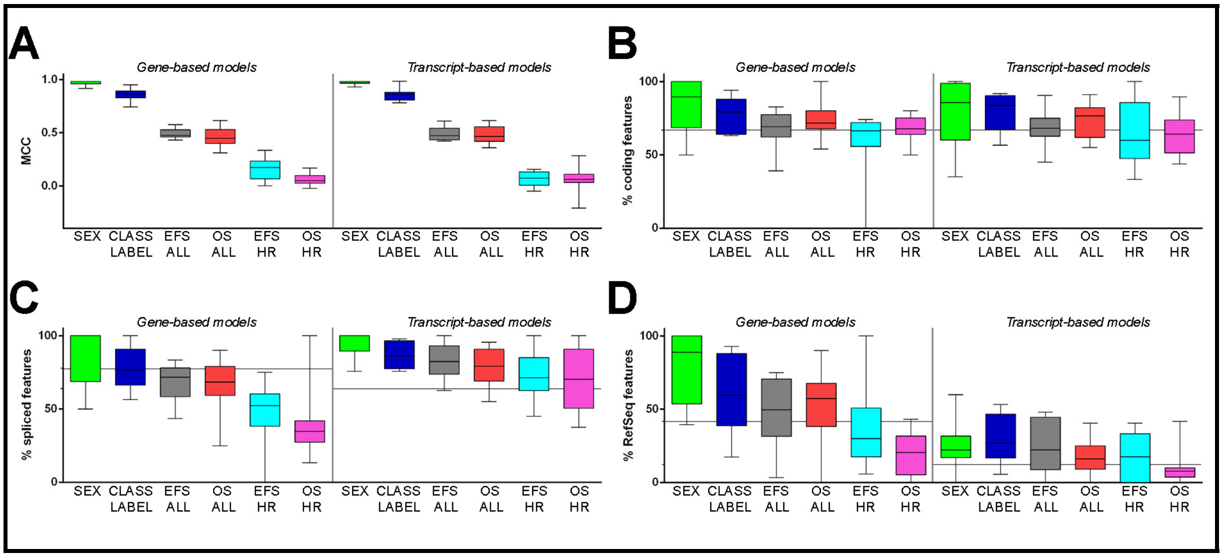

Feature composition of prediction models that were based on the AceView database (MAV and TAV models). (**A**) Prediction performance of MAV and TAV models in terms of MCC. Composition of the MAV and TAV models with regard to (**B**) protein coding features, (**C**) spliced features, and (**D**) content of RefSeq features. Horizontal lines in (B), (C), and (D) indicate the fraction of coding, spliced, and RefSeq features, respectively, as annotated in the AceView database.

Figure S13

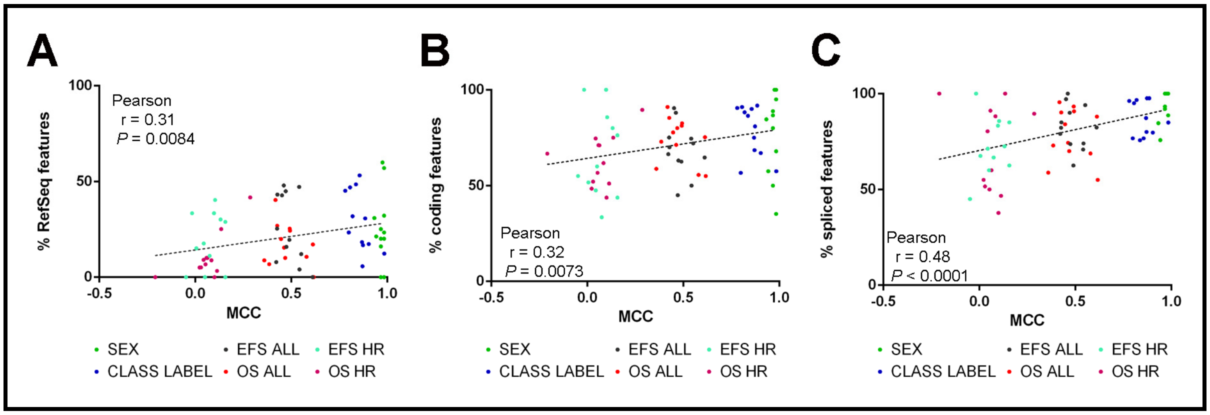

Correlation of prediction performances with the feature composition of prediction models. MCC values of transcript level-based MAV and TAV models were plotted against the fraction of RefSeq features in the model (**A**), the fraction of protein coding features in the model (**B**), and the fraction of spliced features (i.e., genes or transcripts consisting of at least two exons) in the model (**C**).

Part B – Supplementary Tables

Table S1

Definition of 4 major clinico-genetic neuroblastoma subgroups

Subgroup	*MYCN* status	INSS stage	Age at diagnosis	N
MNA	Amplified	Any	≤18 and >18 months	92
ST1	Normal	Stage 1	≤18 and >18 months	117
ST4	Normal	Stage 4	≤18 and >18 months	116
ST4S	Normal	Stage 4S	≤18 months	48

Table S2

Cancer census genes with a complex differential transcript expression pattern in neuroblastoma patients

**Gene**	**Entrez Gene ID**	**Cancer census entry: tumor types**<br>(S: somatic mutations, G: germinal mutations)
GNAS	2778	**S: pituitary adenoma**
TAF15	8148	**S: extraskeletal myxoid chondrosarcoma, acute lymphocytic leukemia**
ASPSCR1	79058	**S: alveolar soft part sarcoma**
FANCG	2189	**G: acute myelogenous leukemia, leukemia**
KTN1	3895	**S: papillary thyroid**
MDM4	4194	**S: glioblastoma, bladder, retinoblastoma**
ZNF331	55422	**S: follicular thyroid adenoma**
AKT2	208	**S: ovarian, pancreatic**
ATM	472	**S: T-cell prolymphocytic leukemia;**<br>**G: leukemia, lymphoma, medulloblastoma, glioma**
CLTC	1213	**S: anaplastic large-cell lymphoma, renal**
ELN	2006	**S: B-cell acute lymphocytic leukemia**
EWSR1	2130	**S: Ewing sarcoma, desmoplastic small round cell tumor, acute lymphocytic leukemia, clear cell sarcoma, sarcoma, myoepithelioma, mesothelioma**
HNRNPA2B1	3181	**S: prostate**
KDM5C	8242	**S: clear cell renal carcinoma**
MLLT6	4302	**S: acute leukemia**
MSH6	2956	**S: colorectal;**<br>**G: colorectal, endometrial, ovarian**
MUTYH	4595	**G: colorectal**
NACA	4666	**S: Non-Hodgkin lymphoma**
NF1	4763	**S: neurofibroma, glioma;**<br>**G: neurofibroma, glioma**
PCM1	5108	**S: papillary thyroid, chronic myeloid leukemia, myeloproliferative disorder**
RAP1GDS1	5910	**S: T-cell acute lymphocytic leukemia**
SFPQ	6421	**S: papillary renal**
TPM3	7170	**S: papillary thyroid, anaplastic large-cell lymphoma, non small cell lung cancer**
TPM4	7171	**S: anaplastic large-cell lymphoma**
TPR	7175	**S: papillary thyroid**
TTL	150465	**S: acute lymphocytic leukemia**

Table S3

Univariate Cox regression analysis of models performing best in terms of MCC in the entire validation set (n=249; endpoints EFS ALL, OS ALL)

Table S3A: Classifiers performing best for event-free survival prediction (EFS)

**Model**	***P* (likelihood ratio test)**	**Hazard Ratio**	**95% CI**
EFS ALL DAT04 MAV T 17	< 0.001	8.968	5.331 to 15.087
EFS ALL DAT03 MAV G 400	< 0.001	7.995	4.854 to 13.170
EFS ALL DAT03 TUC T 800	< 0.001	7.547	4.583 to 12.427
EFS ALL DAT01 AG1 G 38	< 0.001	7.373	4.549 to 11.951
EFS ALL DAT03 AG1 G 9000	< 0.001	5.138	3.290 to 8.023
EFS ALL DAT02 AG1 G 7	< 0.001	4.598	2.991 to 7.069

Table S3B: Classifiers performing best for overall survival prediction

**Model**	***P* (likelihood ratio test)**	**Hazard Ratio**	**95% CI**
OS ALL DAT04 MAV T 20	< 0.001	28.889	11.448 to 72.904
OS ALL DAT03 MAV G 200	< 0.001	26.077	11.100 to 61.264
OS ALL DAT03 TAV T 300	< 0.001	23.218	10.421 to 51.732
OS ALL DAT03 AG1 G 10000	< 0.001	19.658	9.183 to 42.082
OS ALL DAT04 AG1 G 28	< 0.001	24.242	10.289 to 57.116
OS ALL DAT01 AG1 G 40	< 0.001	12.661	6.488 to 24.707

Table S3C: Performance of established prognostic markers

**Model**	***P* (likelihood ratio test)**	**Hazard Ratio**	**95% CI**
Event-free survival			
Stage (4 *v* 1, 2, 3, 4S)	< 0.001	4.694	3.050 to 7.225
MYCN (amplified *vs* normal)	< 0.001	3.131	2.025 to 4.841
Age (>18 m *vs* <18 m)	< 0.001	3.782	2.453 to 5.829
Overall survival			
Stage (4 *v* 1, 2, 3, 4S)	< 0.001	10.978	5.502 to 21.903
MYCN (amplified *vs* normal)	< 0.001	6.710	3.884 to 11.593
Age (>18 m *vs* <18 m)	< 0.001	9.359	4.567 to 19.177

Table S4

Univariate logistic regression analysis of models performing best in terms of MCC in the class-labeled validation set (n=136; endpoint CLASS LABEL)

Model	*P* (likelihood ratio test)	Odds Ratio	95% CI
CLASS LABEL DAT04 MAV T 73	< 0.001	17,942,747	0 to ∞
CLASS LABEL DAT06 MAV G 10	< 0.001	1,958	173 to 22,185
CLASS LABEL DAT02 MAV J 60	< 0.001	631	102 to 3,916
CLASS LABEL DAT03 AG1 G 6000	< 0.001	630	74 to 5,396
CLASS LABEL DAT05 AG1 G 47	< 0.001	261	52 to 1,309
CLASS LABEL DAT02 AG1 G 70	< 0.001	170	42 to 690

Table S5

Univariate Cox regression analysis of models performing best in terms of MCC in the high-risk validation set (n=90; endpoints EFS HR, OS HR)

Table S5A: Classifiers performing best for endpoint E prediction (event free survival)

**Model**	***P* (likelihood ratio test)**	**Hazard Ratio**	**95% CI**
EFS HR DAT06 MAV G 20	0.002	2.297	1.326 to 3.978
EFS HR DAT02 TAV J 120	0.003	2.644	1.303 to 5.367
EFS HR DAT01 TUC T 30	0.023	1.807	1.066 to 3.062
EFS HR DAT02 AG1 G 60	0.016	1.944	1.104 to 3.423
EFS HR DAT03 AG1 G 10000	0.016	1.853	1.106 to 3.104
EFS HR DAT04 AG1 G 3	0.027	1.742	1.068 to 2.842

Table S5B: Classifiers performing best for endpoint F prediction (overall survival)

**Model**	***P* (likelihood ratio test)**	**Hazard Ratio**	**95% CI**
OS HR DAT02 TAV J 71	0.003	2.338	1.305 to 4.190
OS HR DAT06 TAV T 67	0.009	2.150	1.209 to 3.822
OS HR DAT03 TAV J 90	0.002	2.670	1.483 to 4.808
OS HR DAT02 AG1 G 60	0.010	2.116	1.184 to 3.783
OS HR DAT03 AG1 G 10000	***0.221***	---	---
OS HR DAT01 AG1 G 38	***0.592***	---	---

Table S5C: Performance of established prognostic markers

**Model**	***P* (likelihood ratio test)**	**Hazard Ratio**	**95% CI**
Event-free survival			
Stage (4 *v* 1, 2, 3, 4S)	***0.356***	---	---
MYCN (amplified *vs* normal)	***0.903***	---	---
Age (>18 m *vs* <18 m)	***0.827***	---	---
Overall survival			
Stage (4 *v* 1, 2, 3, 4S)	***0.658***	---	---
MYCN (amplified *vs* normal)	***0.233***	---	---
Age (>18 m *vs* <18 m)	***0.329***	---	---

Table S6

Multivariate Cox regression analysis of the single microarray- and RNA-seq-based models which performed best for each endpoint in terms of MCC.

Endpoint / Outcome	Covariables	*P*	Hazard Ratio	95% CI
EFS ALL (n=245)	EFS ALL DAT04 MAV T 17	< 0.001	9.19	5.46 ; 15.48
	Stage	n.s.		
	MYCN	n.s.		
	Age	n.s.		
EFS ALL (n=245)	EFS ALL DAT01 AG1 G 38	< 0.001	5.31	3.02 ; 9.35
	Stage	0.013	1.86	1.12 ; 3.07
	MYCN	n.s.		
	Age	n.s.		
OS ALL (n=245)	OS ALL DAT04 MAV T 20	< 0.001	4.42	2.56 ; 7.65
	Stage	0.014	1.93	1.13 ; 3.30
	MYCN	n.s.		
	Age	n.s.		
OS ALL (n=245)	OS ALL DAT03 AG1 G 10000	< 0.001	2.62	1.52 ; 4.52
	Stage	0.004	2.16	1.26 ; 3.69
	MYCN	n.s.		
	Age	0.081	1.59	0.94 ; 2.71
EFS HR (n=90)	EFS HR DAT06 MAV G 20	0.002	2.30	1.33 ; 3.98
	Stage	n.s.		
	MYCN	n.s.		
	Age	n.s.		
EFS HR (n=90)	EFS HR DAT02 AG1 G 60	0.016	1.94	1.10 ; 3.42
	Stage	n.s.		
	MYCN	n.s.		
	Age	n.s.		
OS HR (n=90)	OS HR DAT02 TAV J 71	0.003	2.34	1.31 ; 4.19
	Stage	n.s.		
	MYCN	n.s.		
	Age	n.s.		
OS HR (n=90)	OS HR DAT02 AG1 G 60	0.010	2.12	1.18 ; 3.78
	Stage	n.s.		
	MYCN	n.s.		
	Age	n.s.		

Table S7

Variance component analysis of model prediction performances

Table S7A: Statistical estimation of the contribution of different factors to the overall variance observed for the prediction model performances as measured by Matthew's correlation coefficient (MCC).

Effect factor	Var Component	% of Total	*p*_Value
Endpoint	0.13184	94.81	**<0.0001**
Platform	0.00000	0.00	0.6042
RNA Seq Pipeline	0.00000	0.00	0.5481
Feature Level	0.00000	0.00	0.1638
Analysis Team	0.00013	0.09	0.0588
Classification Method	0.00000	0.00	0.7229
Log10 (Model Size)	0.00028	0.20	**0.0044**
Platform*Endpoint	0.00000	0.00	0.1835
RNA Seq Pipeline*Endpoint	0.00000	0.00	0.0364
Feature Level*Endpoint	0.00000	0.00	0.0999
Analysis Team*Endpoint	0.00126	0.91	**<0.0001**
Classification Methods*Endpoint	0.00010	0.07	**0.0033**
Log10 (Model Size)*Endpoint	0.00056	0.40	**0.0003**

Table S7B: Best linear unbiased prediction (BLUP) estimates for the 4 factors with significant variance contribution, i.e. log10(model size); endpoint*analysis team, endpoint*classification method, endpoint*log10(model size).

Endpoint	Factor	BLUP Estimate	Std Err Pred	DF	t Value	Pr > |t|
		Log10(Model size)				
overall	Log10(Model size): 1	-0.01800	0.01256	242	-1.43264	0.1533
overall	Log10(Model size): 2	0.00045	0.01208	242	0.03738	0.9702
overall	Log10(Model size): 3	0.01259	0.01258	242	1.00066	0.3180
overall	Log10(Model size): 4	0.00496	0.01309	242	0.37872	0.7052
		Endpoint * Analysis team				
EFS ALL	Analysis Team: 1	0.02208	0.02195	242	1.00608	0.3154
EFS ALL	Analysis Team: 2	0.04625	0.02232	242	2.07183	0.0393
EFS ALL	Analysis Team: 3	-0.07029	0.02139	242	-3.28599	0.0012
EFS ALL	Analysis Team: 4	0.01585	0.02169	242	0.73076	0.4656
EFS ALL	Analysis Team: 5	-0.01979	0.02122	242	-0.93256	0.3520

EFS ALL	Analysis Team: 6	0.00562	0.02096	242	0.26821	0.7888
OS ALL	Analysis Team: 1	0.01274	0.02389	242	0.53325	0.5943
OS ALL	Analysis Team: 2	0.06714	0.02494	242	2.69182	0.0076
OS ALL	Analysis Team: 3	-0.04347	0.02331	242	-1.86473	0.0634
OS ALL	Analysis Team: 4	0.04604	0.02340	242	1.96716	0.0503
OS ALL	Analysis Team: 5	-0.05424	0.02307	242	-2.35118	0.0195
OS ALL	Analysis Team: 6	-0.02855	0.02323	242	-1.22918	0.2202
SEX	Analysis Team: 1	0.01698	0.01862	242	0.91235	0.3625
SEX	Analysis Team: 2	-0.02140	0.01867	242	-1.14637	0.2528
SEX	Analysis Team: 3	0.00175	0.01837	242	0.09545	0.9240
SEX	Analysis Team: 4	-0.01333	0.01862	242	-0.71578	0.4748
SEX	Analysis Team: 5	0.00568	0.01833	242	0.30981	0.7570
SEX	Analysis Team: 6	0.01489	0.01770	242	0.84096	0.4012
Class Label	Analysis Team: 1	-0.00071	0.02116	242	-0.03350	0.9733
Class Label	Analysis Team: 2	0.00697	0.02260	242	0.30859	0.7579
Class Label	Analysis Team: 3	0.00686	0.02096	242	0.32707	0.7439
Class Label	Analysis Team: 4	-0.00617	0.02125	242	-0.29022	0.7719
Class Label	Analysis Team: 5	0.00942	0.02135	242	0.44129	0.6594
Class Label	Analysis Team: 6	-0.01294	0.02074	242	-0.62403	0.5332
EFS HR	Analysis Team: 1	0.00632	0.02683	242	0.23563	0.8139
EFS HR	Analysis Team: 2	-0.00608	0.02730	242	-0.22274	0.8239
EFS HR	Analysis Team: 3	0.02230	0.02677	242	0.83305	0.4056
EFS HR	Analysis Team: 4	-0.03229	0.02764	242	-1.16821	0.2439
EFS HR	Analysis Team: 5	-0.01297	0.02670	242	-0.48560	0.6277
EFS HR	Analysis Team: 6	0.01916	0.02729	242	0.70202	0.4833
OS HR	Analysis Team: 1	-0.00652	0.02738	242	-0.23808	0.8120
OS HR	Analysis Team: 2	-0.00986	0.02740	242	-0.35980	0.7193
OS HR	Analysis Team: 3	0.00471	0.02697	242	0.17482	0.8614
OS HR	Analysis Team: 4	-0.02350	0.02809	242	-0.83635	0.4038
OS HR	Analysis Team: 5	0.01839	0.02686	242	0.68467	0.4942
OS HR	Analysis Team: 6	0.01295	0.02703	242	0.47885	0.6325
Endpoint * Classification algorithm						
EFS ALL	Class.Method: DA	0.00713	0.00981	242	0.72746	0.4676
EFS ALL	Class.Method: GLM	-0.00118	0.01000	242	-0.11810	0.9061
EFS ALL	Class.Method: Logistic	-0.00726	0.00986	242	-0.73662	0.4621
EFS ALL	Class.Method: PLS	-0.00585	0.00958	242	-0.61124	0.5416
EFS ALL	Class.Method: SMO	0.00389	0.00969	242	0.40094	0.6888
EFS ALL	Class.Method: SVM	-0.00310	0.00942	242	-0.32940	0.7421
EFS ALL	Class.Method: Tree	0.00636	0.00927	242	0.68618	0.4933
OS ALL	Class.Method: DA	0.00105	0.00996	242	0.10535	0.9162
OS ALL	Class.Method: Logistic	0.00265	0.01008	242	0.26286	0.7929
OS ALL	Class.Method: PLS	-0.00061	0.00960	242	-0.06358	0.9494
OS ALL	Class.Method: SMO	-0.00198	0.01009	242	-0.19668	0.8442

OS ALL	Class.Method: SVM	0.00195	0.00985	242	0.19804	0.8432
OS ALL	Class.Method: Tree	-0.00308	0.00949	242	-0.32506	0.7454
SEX	Class.Method: DA	-0.00800	0.00784	242	-1.02007	0.3087
SEX	Class.Method: GLM	0.00925	0.00821	242	1.12731	0.2607
SEX	Class.Method: KNN	0.00070	0.00921	242	0.07640	0.9392
SEX	Class.Method: Logistic	0.00070	0.00866	242	0.08037	0.9360
SEX	Class.Method: PLS	0.00000	0.00983	242	-0.00033	0.9997
SEX	Class.Method: SMO	-0.00307	0.00815	242	-0.37679	0.7067
SEX	Class.Method: SVM	0.00203	0.00847	242	0.23927	0.8111
SEX	Class.Method: Tree	-0.00123	0.00791	242	-0.15532	0.8767
Class Label	Class.Method: DA	0.00455	0.00956	242	0.47603	0.6345
Class Label	Class.Method: KNN	-0.00397	0.00962	242	-0.41285	0.6801
Class Label	Class.Method: Logistic	-0.00276	0.00971	242	-0.28375	0.7768
Class Label	Class.Method: PLS	-0.00112	0.00972	242	-0.11553	0.9081
Class Label	Class.Method: SMO	0.00402	0.00963	242	0.41706	0.6770
Class Label	Class.Method: SVM	0.00117	0.00937	242	0.12502	0.9006
Class Label	Class.Method: Tree	-0.00161	0.00943	242	-0.17029	0.8649
EFS HR	Class.Method: DA	0.00343	0.01002	242	0.34201	0.7326
EFS HR	Class.Method: DS	0.00000	0.01020	242	0.00000	1.0000
EFS HR	Class.Method: KNN	-0.00091	0.01015	242	-0.08939	0.9288
EFS HR	Class.Method: Logistic	-0.00248	0.00995	242	-0.24917	0.8034
EFS HR	Class.Method: PLS	-0.00209	0.00997	242	-0.20949	0.8342
EFS HR	Class.Method: SMO	0.00237	0.01011	242	0.23458	0.8147
EFS HR	Class.Method: SVM	0.00133	0.00984	242	0.13553	0.8923
EFS HR	Class.Method: Tree	-0.00195	0.00990	242	-0.19703	0.8440
OS HR	Class.Method: DA	-0.00092	0.01001	242	-0.09184	0.9269
OS HR	Class.Method: DS	-0.00052	0.01015	242	-0.05171	0.9588
OS HR	Class.Method: Logistic	0.00141	0.01004	242	0.14090	0.8881
OS HR	Class.Method: PLS	-0.00019	0.00998	242	-0.01871	0.9851
OS HR	Class.Method: SMO	-0.00146	0.01011	242	-0.14467	0.8851
OS HR	Class.Method: SVM	0.00112	0.00985	242	0.11403	0.9093
OS HR	Class.Method: Tree	0.00024	0.00991	242	0.02445	0.9805
Endpoint * Log10(Model size)						
EFS ALL	Log10(Model size): 1	-0.01236	0.02063	242	-0.59930	0.5495
EFS ALL	Log10(Model size): 2	0.00309	0.01743	242	0.17725	0.8595
EFS ALL	Log10(Model size): 3	0.01483	0.01841	242	0.80582	0.4211
EFS ALL	Log10(Model size): 4	-0.00568	0.01991	242	-0.28539	0.7756
OS ALL	Log10(Model size): 1	-0.04253	0.02002	242	-2.12475	0.0346
OS ALL	Log10(Model size): 2	0.01075	0.01835	242	0.58587	0.5585
OS ALL	Log10(Model size): 3	0.01586	0.01917	242	0.82715	0.4090
OS ALL	Log10(Model size): 4	0.01576	0.02087	242	0.75534	0.4508
SEX	Log10(Model size): 1	0.02888	0.01582	242	1.82598	0.0691
SEX	Log10(Model size): 2	-0.01286	0.01573	242	-0.81768	0.4143

SEX	Log10(Model size): 3	-0.00002	0.01863	242	-0.00095	0.9992
SEX	Log10(Model size): 4	-0.01397	0.01747	242	-0.79931	0.4249
Class Label	Log10(Model size): 1	0.00792	0.01819	242	0.43524	0.6638
Class Label	Log10(Model size): 2	-0.00197	0.01721	242	-0.11456	0.9089
Class Label	Log10(Model size): 3	-0.00642	0.01779	242	-0.36077	0.7186
Class Label	Log10(Model size): 4	0.00199	0.01987	242	0.10033	0.9202
EFS HR	Log10(Model size): 1	-0.00940	0.02011	242	-0.46742	0.6406
EFS HR	Log10(Model size): 2	-0.00138	0.01974	242	-0.06980	0.9444
EFS HR	Log10(Model size): 3	-0.00083	0.02188	242	-0.03815	0.9696
EFS HR	Log10(Model size): 4	0.01003	0.02208	242	0.45457	0.6498
OS HR	Log10(Model size): 1	-0.00786	0.02107	242	-0.37295	0.7095
OS HR	Log10(Model size): 2	0.00326	0.01988	242	0.16388	0.8700
OS HR	Log10(Model size): 3	0.00131	0.02075	242	0.06307	0.9498
OS HR	Log10(Model size): 4	0.00160	0.02237	242	0.07142	0.9431

Table S7C: Direct pairwise statistical comparison of the platform, the pipeline and feature levels as factors influencing the prediction model performance, yet without significant variance contribution.

Endpoint Cohort	Label	Estimate	Standard Error	DF	t Value	Pr > |t|
EFS ALL	*Platform:* Microarray vs. RNA-seq	-0.04618	0.02206	242	-2.09337	**0.0374**
EFS ALL	*Pipeline:* MAV vs. TAV	0.01739	0.01575	242	1.10473	0.2704
EFS ALL	*Pipeline:* MAV vs. TUC	0.01592	0.01462	242	1.08888	0.2773
EFS ALL	*Pipeline:* TAV vs. TUC	-0.00147	0.01553	242	-0.09482	0.9245
EFS ALL	*Feature Level:* Gene vs. Junction	0.03022	0.01664	242	1.81648	0.0705
EFS ALL	*Feature Level:* Gene vs. Transcript	0.00420	0.01429	242	0.29411	0.7689
EFS ALL	*Feature Level:* Junction vs. Transcript	-0.02602	0.01693	242	-1.53667	0.1257
OS ALL	*Platform:* Microarray vs. RNA-seq	-0.01046	0.02935	242	-0.35639	0.7219
OS ALL	*Pipeline:* MAV vs. TAV	0.01480	0.02019	242	0.73300	0.4643
OS ALL	*Pipeline:* MAV vs. TUC	-0.00653	0.02029	242	-0.32184	0.7479
OS ALL	*Pipeline:* TAV vs. TUC	-0.02133	0.02024	242	-1.05379	0.2930
OS ALL	*Feature Level:* Gene vs. Junction	0.05176	0.02206	242	2.34604	**0.0198**
OS ALL	*Feature Level:* Gene vs. Transcript	0.01254	0.02135	242	0.58706	0.5577
OS ALL	*Feature Level:* Junction vs. Transcript	-0.03922	0.02153	242	-1.82214	0.0697
SEX	*Platform:* Microarray vs. RNA-seq	0.00928	0.00957	242	0.97033	0.3328
SEX	*Pipeline:* MAV vs. TAV	0.00078	0.00741	242	0.10477	0.9166
SEX	*Pipeline:* MAV vs. TUC	0.00583	0.00702	242	0.83072	0.4069
SEX	*Pipeline:* TAV vs. TUC	0.00505	0.00736	242	0.68637	0.4931
SEX	*Feature Level:* Gene vs. Junction	-0.01058	0.00810	242	-1.30559	0.1929
SEX	*Feature Level:* Gene vs. Transcript	-0.00593	0.00688	242	-0.86175	0.3897

SEX	*Feature Level:* Junction vs. Transcript	0.00464	0.00772	242	0.60204	0.5477
Class Label	*Platform:* Microarray vs. RNA-seq	-0.00807	0.02521	242	-0.32009	0.7492
Class Label	*Pipeline:* MAV vs. TAV	0.03530	0.01630	242	2.16499	**0.0314**
Class Label	*Pipeline:* MAV vs. TUC	0.03609	0.01619	242	2.22992	**0.0267**
Class Label	*Pipeline:* TAV vs. TUC	0.00080	0.01620	242	0.04914	0.9609
Class Label	*Feature Level:* Gene vs. Junction	-0.00495	0.01873	242	-0.26409	0.7919
Class Label	*Feature Level:* Gene vs. Transcript	0.00726	0.01579	242	0.45949	0.6463
Class Label	*Feature Level:* Junction vs. Transcript	0.01220	0.01782	242	0.68479	0.4941
EFS HR	*Platform:* Microarray vs. RNA-seq	0.02839	0.04812	242	0.59010	0.5557
EFS HR	*Pipeline:* MAV vs. TAV	-0.01607	0.03761	242	-0.42716	0.6696
EFS HR	*Pipeline:* MAV vs. TUC	-0.01323	0.04058	242	-0.32609	0.7446
EFS HR	*Pipeline:* TAV vs. TUC	0.00283	0.03454	242	0.08204	0.9347
EFS HR	*Feature Level:* Gene vs. Junction	0.07744	0.04019	242	1.92666	0.0552
EFS HR	*Feature Level:* Gene vs. Transcript	0.06053	0.03709	242	1.63193	0.1040
EFS HR	*Feature Level:* Junction vs. Transcript	-0.01691	0.03880	242	-0.43584	0.6633
OS HR	*Platform:* Microarray vs. RNA-seq	0.07153	0.05440	242	1.31482	0.1898
OS HR	*Pipeline:* MAV vs. TAV	-0.11794	0.03500	242	-3.36925	**0.0009**
OS HR	*Pipeline:* MAV vs. TUC	-0.06882	0.03650	242	-1.88571	0.0605
OS HR	*Pipeline:* TAV vs. TUC	0.04911	0.03733	242	1.31570	0.1895
OS HR	*Feature Level:* Gene vs. Junction	-0.02079	0.03692	242	-0.56326	0.5738
OS HR	*Feature Level:* Gene vs. Transcript	-0.01952	0.03514	242	-0.55539	0.5791
OS HR	*Feature Level:* Junction vs. Transcript	0.00127	0.03458	242	0.03687	0.9706

Table S8

Correlation of prediction performances and the feature composition with regard to the fraction of RefSeq, protein coding, and spliced features in MAV and TAV models

Correlation of prediction performances (in terms of MCCs in the validation cohort) and the feature composition of MAV and TAV models with regard to the fraction of RefSeq features in the model (**a**, gene level; **b**, transcript level), the fraction of protein coding features in the model (**c**, gene level; **d**, transcript level), and the fraction of spliced features (i.e., genes or transcripts consisting of at least two exons) in the model (**e**, gene level; **f**, transcript level).

(A) Gene-based: RefSeq features

	SEX ALL	CLASS LABEL	EFS ALL	OS ALL	EFS HR	OS HR	overall
**%-RefSeq (median)**	88.89	59.65	49.65	57.36	30.01	20.56	46.59
**Pearson r**	0.7	-0.053	0.072	-0.31	0.029	0.18	0.6
**95% confidence interval**	0.21 to 0.91	-0.61 to 0.54	-0.52 to 0.62	-0.75 to 0.32	-0.55 to 0.59	-0.44 to 0.68	0.43 to 0.73
**R squared**	0.49	0.0028	0.0052	0.097	0.00083	0.032	0.36
***P* value**	0.0111	0.8703	0.8242	0.3242	0.9291	0.5766	< 0.0001

(B) Transcript-based: RefSeq features

	SEX ALL	CLASS LABEL	EFS ALL	OS ALL	EFS HR	OS HR	overall
**%-RefSeq (median)**	22.27	26.96	22.36	16.18	17.5	7.745	18.33
**Pearson r**	0.087	-0.58	-0.44	-0.27	0.25	0.73	0.31
**95% confidence interval**	-0.51 to 0.63	-0.87 to -0.0089	-0.81 to 0.17	-0.73 to 0.36	-0.41 to 0.74	0.26 to 0.92	0.083 to 0.51
**R squared**	0.0076	0.34	0.2	0.075	0.065	0.53	0.096
***P* value**	0.7876	0.0481	0.1487	0.3903	0.4503	0.0073	0.0084

(C) Gene-based: Coding features

	SEX ALL	CLASS LABEL	EFS ALL	OS ALL	EFS HR	OS HR	overall
**%-Coding (median)**	89.45	79.3	69.21	71.7	66.37	67.85	70.3
**Pearson r**	0.76	-0.066	0.19	-0.77	0.038	0.25	0.41
**95% confidence interval**	0.33 to 0.93	-0.62 to 0.53	-0.43 to 0.69	-0.93 to -0.35	-0.55 to 0.60	-0.38 to 0.72	0.19 to 0.58
**R squared**	0.57	0.0044	0.038	0.59	0.0014	0.061	0.17
***P* value**	0.0043	0.8378	0.5444	0.0033	0.9074	0.4395	0.0004

**(D)** Transcript-based: Coding features							
	**SEX ALL**	**CLASS LABEL**	**EFS ALL**	**OS ALL**	**EFS HR**	**OS HR**	**overall**
**%-Coding (median)**	85.65	83.72	68.25	76.58	60	64.22	72.97
**Pearson r**	0.14	-0.48	-0.25	-0.36	0.072	0.31	0.32
**95% confidence interval**	-0.47 to 0.66	-0.82 to 0.14	-0.72 to 0.38	-0.77 to 0.27	-0.55 to 0.64	-0.32 to 0.75	0.089 to 0.51
**R squared**	0.019	0.23	0.061	0.13	0.0051	0.098	0.1
***P* value**	0.6656	0.1181	0.439	0.2523	0.8342	0.3226	0.0073

**(E)** Gene-based: Spliced features							
	**SEX ALL**	**CLASS LABEL**	**EFS ALL**	**OS ALL**	**EFS HR**	**OS HR**	**overall**
**%-Spliced (median)**	100	76.59	71.67	68.34	52.18	34.77	67.21
**Pearson r**	0.76	-0.11	0.2	-0.039	-0.22	0.039	0.65
**95% confidence interval**	0.32 to 0.93	-0.65 to 0.49	-0.42 to 0.70	-0.60 to 0.55	-0.70 to 0.41	-0.55 to 0.60	0.49 to 0.76
**R squared**	0.57	0.013	0.041	0.0015	0.047	0.0015	0.42
***P* value**	0.0045	0.7247	0.526	0.9046	0.4984	0.9041	< 0.0001

**(F)** Transcript-based: Spliced features							
	**SEX ALL**	**CLASS LABEL**	**EFS ALL**	**OS ALL**	**EFS HR**	**OS HR**	**overall**
**%-Spliced (median)**	100	86.13	82.28	79.23	71.25	70.2	84.62
**Pearson r**	0.75	-0.16	-0.18	-0.11	0.23	-0.092	0.48
**95% confidence interval**	0.31 to 0.92	-0.67 to 0.46	-0.68 to 0.44	-0.64 to 0.50	-0.43 to 0.73	-0.63 to 0.51	0.28 to 0.65
**R squared**	0.56	0.024	0.031	0.012	0.054	0.0084	0.23
***P* value**	0.0051	0.6281	0.5832	0.7379	0.4927	0.7766	< 0.0001

Table S9

Classification algorithms selected by data analysis teams.

Classification Algorithm	No. of DATs[1]	No. of models
Distance Scoring	1	2
Discriminant Analysis	3	97
General Linear Model	2	4
K-Nearest Neighbor	1	6
Nearest Centroid	1	15
Logistic Regression	2	19
Partial Least Squares	3	30
Sequential Minimal Optimization	1	17
Support Vector Machine	3	120
Radial Basis Machine	1	18
Decision Trees	2	32

[1]*DATs, data analysis teams using the specified algorithms*

Part C – Supplementary Notes

SUPPLEMENTARY NOTE 1: *The Magic RNA-seq Analysis Pipeline*

The Magic RNA-seq analysis pipeline (developed by Danielle Thierry-Mieg & Jean Thierry-Mieg at the NCBI/NIH, Bethesda, MD) has been part of many SEQC studies and been detailed previously ([3, 4], Fig SN1). A brief description of its application for the mapping and quantification of the SEQC neuroblastoma sequencing data is given below.

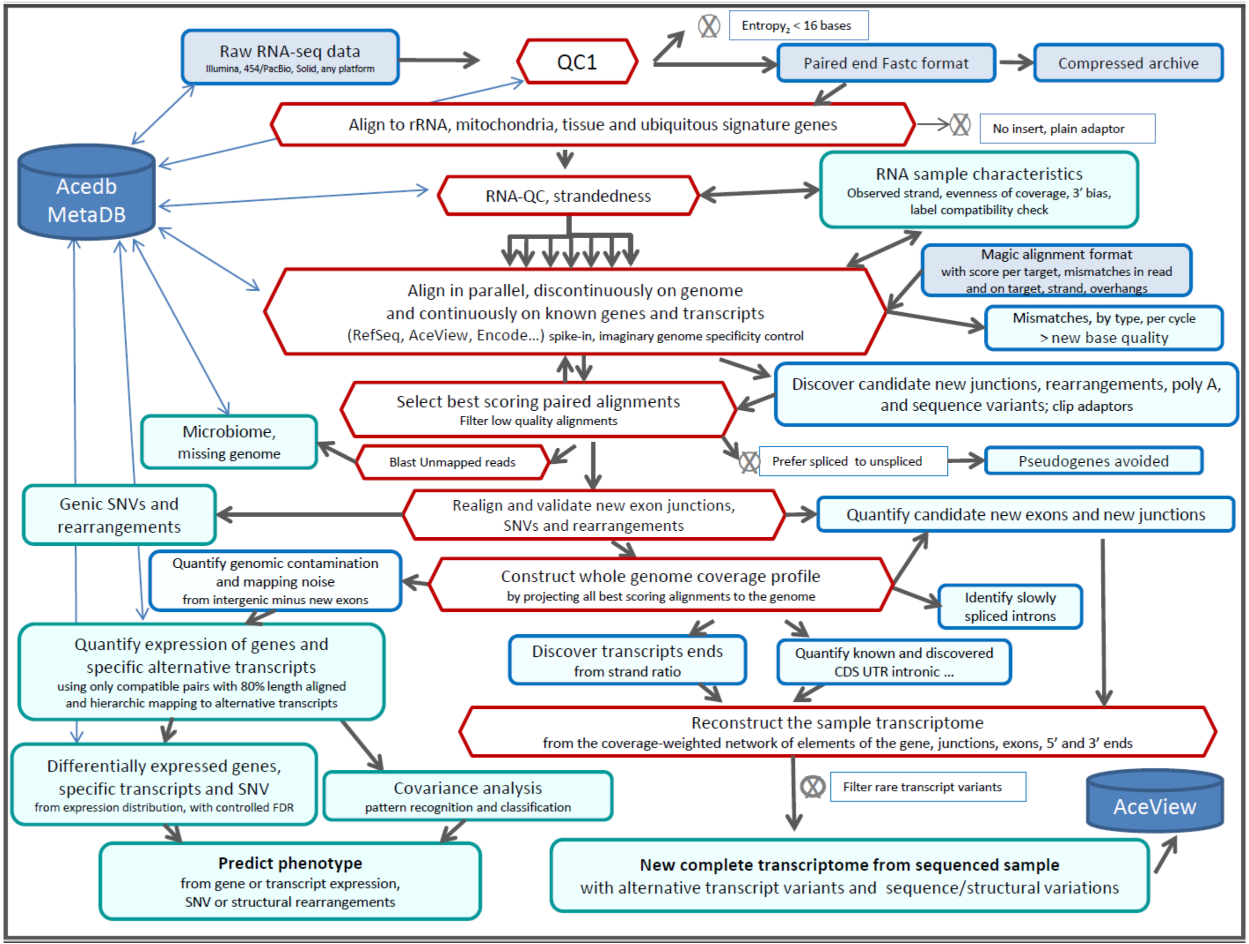

Figure SN1: *The complete Magic pipeline for RNA-seq analysis. The Magic code is available on the AceView NCBI website in the Downloads / Software tab / Magic, i.e. at ftp://ftp.ncbi.nlm.nih.gov/repository/acedb/Software/Magic. The package, written in C, is completely original, self-standing and open source. It includes its own aligner and software to perform the integrative data analysis presented here, and generates expression data tables as well as multiple quality control reports. Typically, one gigabase of RNA sequences are aligned on all targets using about 6 hours on a single CPU, 8 GB of RAM and 2 gigabytes of disk space and the entire Neuroblastoma project runs in parallel overnight on the NCBI farm. In practice there are no parameters to specify as Magic is auto-adaptive. The experimental details for each run are entered in the species-specific object oriented AceView/AceDB run database, essentially locating the original fastq file and specifying the platform (Illumina, Roche, SOLiD, PGM/Proton, PacBio, Helicos), the protocol (total or polyA, small RNA or not; stranded protocol or not) and the eventual existence of paired reads. Relevant groups of runs are manually created in the database, for instance in the SEQC study, by sample, platform, site or library, and in this NB study, by phenotypes, or stages, training and test. Actions to be performed such as expression index measures, transcript discovery, SNP detection, sample comparisons, DEG calls or covariance analysis, titration or serial profiling analysis and so on are manually specified for each group. The pipeline is then run very simply for example as: "MAGIC ALIGN GENE_EXPRESSION INTRON_DISCOVERY SNP".*

SN1.1 Mapping

A summary of the clinical and experimental details, with statistics on QC and mapping results for each sample, is provided in Supplementary file 4 [xls]. The mapping target sequences for the SEQC projects, available as Targets/hs.*.fasta.gz at:

ftp://ftp.ncbi.nlm.nih.gov/repository/acedb/Software/Magic/TARGETS/SEQC.TARGET.human.2013_06_15.tar.gz, consist of

- the reference genome (GRCh37, without patches)
- AceView genes (264,387 transcripts, from 55,836 genes in AceView 2011, which is a subset of the 2010 version where more single exon genes were moved to the ‘cloud’ class): AceView 2010 genes summarize the 9 million human cDNA sequences in GenBank, and RefSeq/Gene NM/NR transcript models from 2010 (37.1). The cDNAs define about twice as many genes and close to twice as many exon junctions than RefSeq only (370,000 rather than 198,000). AceView 2010 and 2011 annotate 205,691 spliced (multi-exon) transcript variants. Entrez Gene names are used when available; otherwise genes are named using an AceView identifier, stable across releases.
- RefSeq v104 (40,894 transcripts from 24,536 genes, January 2013)
- Gencode v15 / Ensembl 37.70 (cdna.all and ncRNA, minus rRNAs: 194,703 transcripts from 56,071 genes, released January 2013).
- the mitochondrial genome (NC_012920.1),
- ribosomal RNA 5S and 45S precursor (NR_046235 and NR_023363),
- small non coding RNA genes (including 8,611 genes from Ensembl 37.70: 1,923 snRNA, 1,529 snoRNA, 3,110 miRNA and 2,049 misc-RNA, complemented by 625 tRNAs from Lowe, UCSC tRNA track, February 2012),
- the 92 ERCC RNA spike-in sequences
- finally, an imaginary genome used as a mapping specificity control. The imaginary whole genome is constructed by complementing the bases (exchange A:T and G:C), but not reversing the order: it has exactly the same statistics as the genome but is a completely alien decoy. Hits to this target are false positives and their characteristics are used to tune the alignment quality filters (minimum length aligned and maximum mismatch rate).

Regarding transcript expression, distinctive parts of each alternative transcript are measured. This is achieved by a hierarchic mapping approach as illustrated in Figure SN2 on the example of gene UTP14A. Each read mapping equally well to multiple transcripts (e.g. in exons common to transcript variants .a, .b and .c) is attributed to the variant with the first alphabetic name (here variant .a). (Note: AceView transcripts are named by decreasing order of protein coding potential or of length for non-coding genes).

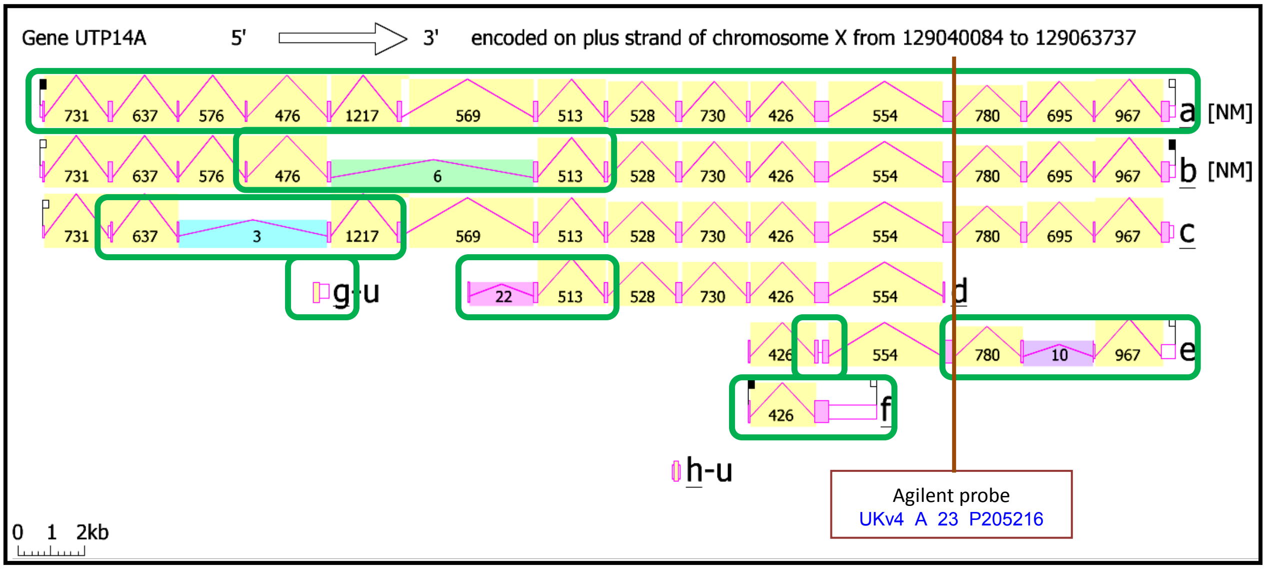

Figure SN2. *Illustration of the 'hierarchic mapping' method used to gain a uniquely specific measure of alternative variants in a gene. On this copy of the AceView gene diagram for UTP14A (top page on www.aceview.org), the alternative variants, with their names to the right (a b …h) and eventual inclusion of RefSeq transcripts ([NM] for coding, [NR] for non-coding) are shown from 5' to 3'. Exons are at the indicated scale (bottom left), and when known, the observed 5' and 3' ends of transcripts are indicated by the 5' and 3' flags (dark for strong evidence, light for less strong; black for canonical AATAAA signal, blue for other signals). Introns are not at scale, but appear in the order they do on the genome. Introns shared between transcripts have the same highlight color (yellow for all introns in variant .a or shared with .a); different colors, here green, blue, pink and purple, identify alternative splicing, and are actually the features distinguishing variants b, c, d and e respectively. The number of supporting cDNA reads for each exon junction in 2 large scale RNA-seq experiments (Pilot SEQC main, A and B samples) and in GenBank/dbEST are indicated. The position of the Agilent probe for this gene, which maps to an exonic sequence common to variants a, b, c and e, is indicated. When comparing differential probes and transcripts, a differential probe indicates the area of the gene is differential, but does not imply that each transcript it maps to is specifically differential. In contrast, when using the hierarchic method, the special features of each transcript, boxed in green, are the only regions attributed to the transcript (note that the boxes spread to nearby exons to reflect the insert length, in the 100-300 bp range). Each transcript will therefore appear differential if and only if its green-boxed regions are expressed differentially: alternative promoters and first exons, alternative last exons and polyA selection sites, and alternative splice patterns are systematically and specifically captured.*

As a consequence, if two transcripts, such as .a and .b, share 2.4 kb in 14 common exons but differ only by one splice junction skipping a cassette exon, only the reads mapping specifically to this structural difference (characteristic of transcript .b, but absent from .a) will score in .b. Similarly, only the reads mapping to the extended alternative 3'UTR part in transcript .f and not present in any of the variants .a .b .c .d .e will score in .f. Using the simple rule of hierarchic attribution of the reads (exemplified in Figure SN2), the differential use of a particular promotor, termination signal or an alternative splice pattern will be associated systematically and specifically to the first variant possessing this feature. Another advantage is that the method is numerically stable, as it does not involve a matrix inversion, and is valid even if additional alternative forms, not yet annotated, exist *in vivo*.

SN1.2 Quantification of RNA-seq expression data

In each gene, the number of reads and bases aligned are collected and used to compute the expression index. Other indexes measuring the properties of each gene in the conditions of the experiment are stored: i) the number of mismatches per kilobase aligned per gene, which points, by comparison to other projects, to project-specific mutation rates, variable genes or pseudogenes, ii) the percentage of orphans and partially aligned reads, which informs on incomplete annotation or rearrangements in the gene, iii) the

percentage of reads mapping non-uniquely to the gene, which informs on gene families and mapping ambiguities.

The Magic normalized RNA-seq index of expression is directly comparable to a normalized microarray logarithmic signal. The logarithm is introduced because gene expression is mostly log-normal. As in FPKM, the index is normalized to be independent of the size of the experiment and of the length of the gene. But several corrections are quantified run by run and introduced to compensate for undesirable batch effects: i) the insert length of the library, which introduces a steric constraint on the coverage of short genes; ii) the 3' bias, which limits the coverage of long genes; iii) the level of genomic contamination, sequencing and mapping noise, estimated from the density of intergenic reads outside new exons discovered in the experiment; this non-specific noise needs to be subtracted; iv) the eventual presence of extremely highly expressed genes, such as albumin in liver or hemoglobins in blood. Correcting for these measurable effects, which are library dependent and occasionally strong, stabilizes the Magic index across samples. Finally, the sampling fluctuations, which affect the counts in low expressed genes, are somewhat absorbed by the log(sqrt) function, also known as inverse hyperbolic sine. For genes that are not 'significantly expressed', i.e. with less than 4 reads above the intergenic noise, the index value is flagged with by an 'NA' (Not Accurate) or 'NE' (Not Expressed, i.e. in case of zero counts), indicating a tradeoff between low precision due to high sampling error and low accuracy due to the proximity of the zero counts plateau.
All these corrections affect the normalization factor z, defined below, while maintaining the read counts n on the original scale, to correctly control the sampling fluctuations. Neglecting these refinements and corrections, the Magic index would be equal to $\log_2$(1000 FPKM), and a corrected FPKM or 'significant' FPKM (sFPKM) of 1 corresponds to an index of 9.96.

The index, resulting from optimization using the SEQC data, is computed as follows:

$$\text{Index} = \log_2 \left(n+\sqrt{G+4b^2+n^2}\right)-1 + \log_2(z), \text{ with } z = 10^{12} r/N'l'$$

$$\text{sFPKM} = 2^{\text{Index}} / 1000, \text{ i.e. Index} = \log_2(1000 \text{ sFPKM})$$

- n is the number of reads aligned in the gene. A read partially aligned counts as a fraction.
- b is the intergenic background noise density multiplied by the gene length.
- r is the average aligned length of the reads, in bases.
- $N'/10^{12}$ is the corrected number of terabases aligned to the known transcriptome, after excluding the ribosomal and mitochondrial genes and the genes gathering more than 2% of the total number of bases aligned (31 instances among the 498 neuroblastoma samples, affecting 16 times the gene EEF1A1, 7 times DDX1, 4 times ALB, 2 times COL1A1, and once COL1A2 or TRIB2).

- l' is the length of the gene effectively sequenceable in the particular experiment. It is influenced by the 3' bias, and the length with 50% combined drop is used in each run as an upper bound l_{max}. Second, the average length λ of the insert is subtracted from the length l of each gene to compensate for the steric constraint. Indeed $l'=(l - \lambda)$ is the number of possible positions of the insert in the gene, and this correction matters for short genes, under-represented in libraries with long insert size. Finally, l' is restricted to the interval $[\lambda, l_{max}]$.
- If the number n of reads in the gene is large, the index is approximately $\log_2(z .n)$ representing the $\log_2$ of the coverage of the gene per terabase aligned.

When the same biological sample is sequenced in multiple runs (15 NB samples had duplicate runs), reads mapped to the same gene are cumulated before computing the index. In phenotypic groups consisting of different individuals, the index of the group is computed as the average index of all participants. Thus in additive (single sample) groups, we compute the log of the average of the number of reads, while in diverse phenotypic groups, we compute the average of the log.
In the favored mode ensuring high specificity of the mapping (used for the differential gene expression analysis per disease stage), only uniquely and quasi-fully aligned 'compatible' pairs are used to compute the expression indexes. The MAV expression tables provided for the AceView Magic predictions used a less stringent version of the expression index, allowing quasi unique alignments and some partial. Transcripts were not hierarchic, and a different quantification of the zero counts was used: the index corresponding to read counts below 4 were linearly interpolated between 0 for 0 reads and the computed index value for 4 reads. All expression tables are sorted by descending maximal index of expression across the 498 samples. The first columns contain metadata connecting AceView genes to RefSeq IDs, Entrez GeneIDs, Agilent expression array probes, and genomic coordinates based on the human reference build 37.

<u>SN1.3 Analysis of the annotated transcriptome</u>

The MAGIC method was applied to evaluate the complexity of deeply sequenced transcriptomes, and count how many previously annotated genes, specific alternative transcripts, and exons junctions appear significantly expressed (not NA/NE flagged). Magic was homogeneously applied to 498 neuroblastoma samples and 2,352 replicate measures of the four RNA samples from SEQC main study ([5]; A, Agilent/Stratagene UHR mixed cultured cells; B, Ambion brain, from various brain regions from 23 donors; C and D, ¾ ¼ and ¼ ¾ mixtures of A and B, respectively), done at 12 laboratory sites using either Illumina HiSeq, Lifetech SOLiD, or Roche 454Ti. The two projects were of similar size and 5.69 terabases of RNA-seq were mapped. All measures were uniformly generated using the stringent unique mode, with hierarchical transcripts, and mapping on the same genome (37) and same gene annotations: RefSeq v104 (2013), Gencode v15/Ensembl v37.70 (2013), and AceView 2011.

RNA-seq experiment description and size	Protocol
SEQC B: Ambion Brain, 0.683 Tb aligned: ILM 395 runs 646 Gb aligned, LIF 190 runs 35.4 Gb, Roche 6 runs 1.25 Gb	polyA non stranded PE 100+100 ILM and polyA stranded PE 50+35 Solid and Roche Titanium
SEQC A: 0.675 Tb aligned, ILM 634 Gb, LIF 39.4 Gb, Roche 1.25 Gb	polyA non stranded PE 100+100 ILM and polyA stranded PE 50+35 Solid and Roche Titanium
SEQC D: 0.673 Tb aligned: ILM 634 Gb + LIF 39.0 Gb	polyA non stranded PE 100+100 ILM and polyA stranded PE 50+35 Solid
SEQC C: 0.682 Tb aligned: ILM 645 Gb + LIF 36.8 Gb	polyA non stranded PE 100+100 ILM and polyA stranded PE 50+35 Solid
**SEQC Main A+B+C+D: 2.716 Tb aligned (2352 runs)**	polyA non stranded PE 100+100 ILM and polyA stranded PE 50+35 Solid and Roche Titanium
**Neuroblastoma: 498 samples, 2.97 Tb aligned (513 runs)**	polyA non stranded PE 100+100 ILM

Annotated and expressed in SEQC RNAseq	<-----------Genes------------->			<---------Transcripts---------->			<--------------Exon Junctions----------------->			
	RefSeq	Gencode	AceView	RefSeq	Gencode	AceView	RefSeq	Gencode	AceView	RefSeq Encode AceView
Annotated	24536	56071	55836	40894	194703	264387	207542	345433	383105	417816
Brain B	19297	26053	36958	29598	91543	135867	186466	256441	281453	289340
Cells A (UHR)	19580	26976	38083	29913	96000	142655	186892	259979	286988	295105
D: 3/4 Brain + 1/4 cells	19958	27487	39091	30894	98313	145712	191281	268408	296525	305268
C: 3/4 cells + 1/4 Brain	19981	27598	39617	30911	99205	147826	191310	269348	297888	306773
SEQC main A + B + C + D (2.716 Tb)	20603	30593	43219	32541	115363	171346	196929	289261	322919	334885
Neuroblastoma (2.97 Tb)	21499	36723	48415	34444	138540	204352	196320	286300	319231	330660

Table SN1: *Complexity of the transcriptome measured homogeneously by Magic. Upper panel: samples, platform, size of experiment and library preparation protocols. Lower panel: counts of annotated and significantly expressed genes, transcripts, exon junctions in each of the 3 annotations. The last column shows the union of unique junctions from either annotation.*

Counts of significantly expressed genes, transcripts and exon junctions indicate that sample A (UHR) expresses a richer variety of features than sample B (brain), and as expected the D and C mixes in this order are even more complex. The union of ABCD comes next, followed by the neuroblastoma cohort, where not surprisingly the 498 independent samples express a greater variety of all features.

SN1.4 Discovery of novel introns and exons

Magic has a built-in ability to discover new exon junctions, inherited from its predecessor, the AceView aligner. Key to the RNA-seq version is the scoring system, which clips the alignments of the reads on perfectly matching zones with at least 8 exact bases on each side (Figure SN3).

Memorizing the score of the alignments (1 point per base aligned minus 8 points per mismatch) allows to recursively optimize the construction of a better transcriptome, including new structural features (new genes, new exon junctions, new rearrangements and new SNPs), by identifying, in the validation phase, which reads align better (with increased score) on the new structures.

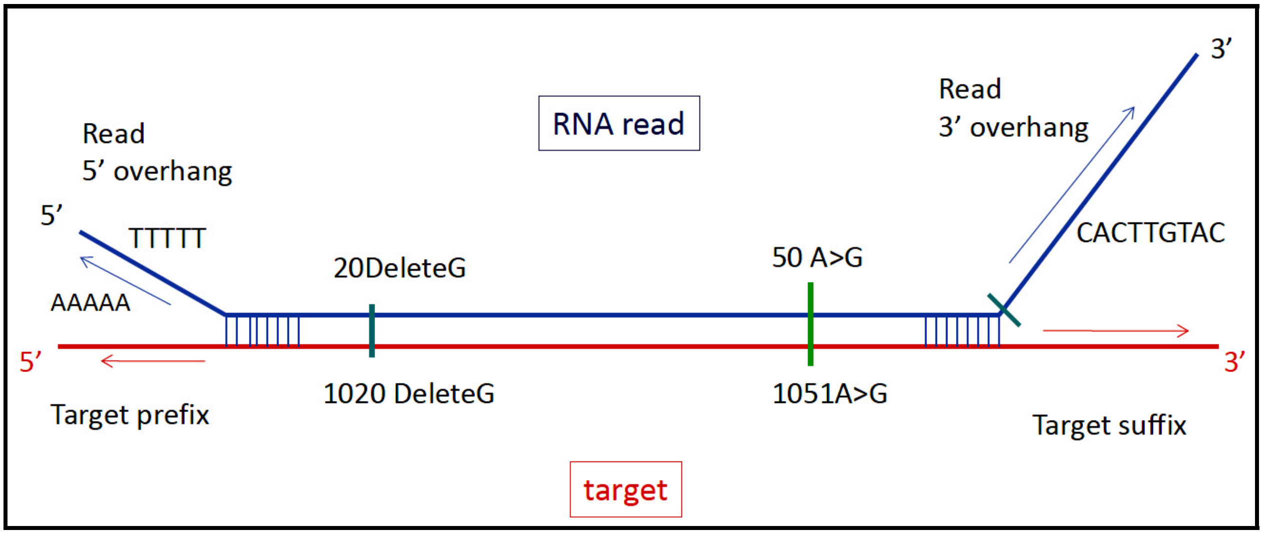

Figure SN3. *The Magic alignment format captures explicitly all useful features of the read-to-target(s) alignment: score, coordinates of alignment ends, length aligned, length to align (after removal of recognized adaptors and polyA), orientation, overhanging sequences and mismatches in read and target coordinates (altogether 22 columns). This comprehensive description avoids the need to re-interpret the data in downstream analyses. All alignments of a given fragment on the genome and on transcripts – always associated to their gene - come on consecutive lines, used together to report the number of distinct alignment sites. If the library is non-stranded (as is the case here), all reads mapping to a single site transcribed in antisense from two genes are flagged. The overhanging (unaligned) sequences are recorded from the end of the alignment outwards (i.e. on the strand of the read on its 3’ side and on the opposite strand on its 5’ side). Finally, the format reports the topological status and distance between read pairs which is exploited when describing chromosomal rearrangements.*

Thanks to the scoring system, alignments from different reads reaching a discontinuity (often an intron) are interrupted at the exact same base, and the consensus of the overhang is readily available, allowing to cooperatively find the other end of the discontinuity by complementarity with the nearby genome (de uno method) or with matching overhangs (de duo). This procedure does not use knowledge of the donor/acceptor consensus; hence it also recognizes deletions, insertions or larger rearrangements as well as non-canonical splice sites. If the reads are long (for human, >75 bp), most exon junctions are discovered by analyzing the discontinuous alignment of individual reads (de uno method). For shorter read lengths (down to 35 bp), the cooperative de duo method is needed, but can be efficient too. Cases where the overhanging sequence matches the adaptor are clipped, a useful feature for analyzing small RNAs or short insert libraries, for which the read length might be longer than the transcripts. This technique of dynamical clipping during alignment is more sensitive and more specific than clipping the reads before alignment. Simpler cases, in which the overhanging sequence matches a polyA, are reported and evaluated cooperatively to eventually flag the 3’ ends of transcripts.

Analysis of the coverage plots is used for quantification of exons and sub-segments and to discover new candidate exons. Alignments to the various targets are merged by unique projection on the genome. Fragments overlapping an exon junction in a transcript or novel candidate junctions are cleanly split by projection into multiple exons, yielding a coverage plot with sharp exon boundaries and no unsupported leak into introns.

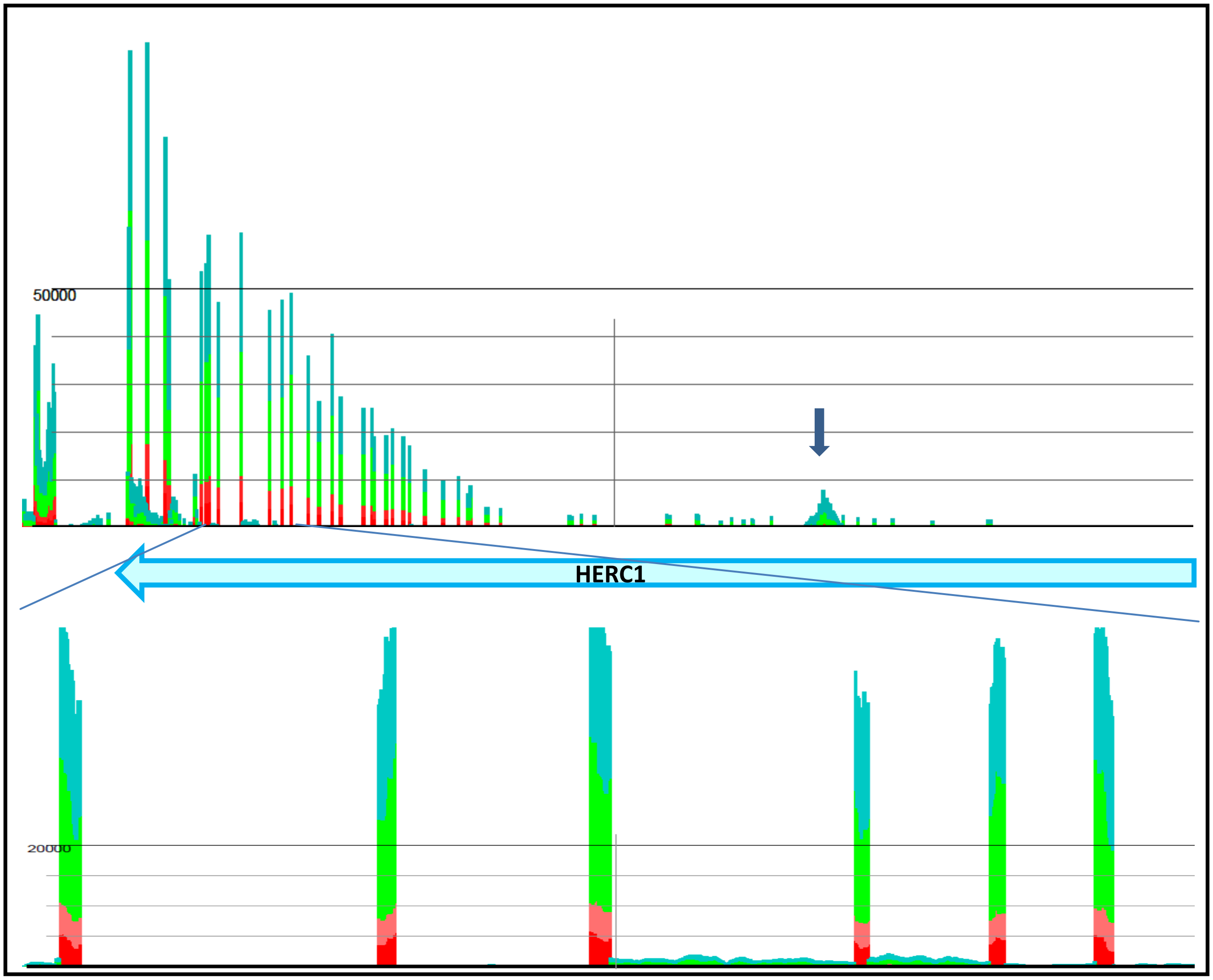

Figure SN4*: Typical example of coverage plot showing all aligned bases (no erasing), yet exons are detected with sharp boundaries. The HERC gene, with its 92 introns annotated in AceView, extends over 225 kb on the genome, on the reverse strand (blue arrow, 5' on the right). The vertical scale indicates the number of reads covering each position of the genome. The green represents the contribution from 392 individuals who survived and the red from 106 who did not. The light and dark colors tones show the forward and reverse reads. Limited 3' bias is observed along the 15.2 kb transcript, from ~1000 fold coverage in first exon to 100,000 fold in last 3' exon. Notice an 8 kb volcano-like intronic sequence (arrow) in a region not conserved in Macaca and other mammals appears expressed. Zooming in on 6 central exons (spread over 10 kb) allows appreciating the slower rate of splicing, or the increased stability of some intronic sequences (2 introns central in the picture). This phenomenon is commonly observed for specific introns in many genes, suggesting possible regulatory role of the splicing dynamics.*

To ensure stringency, the main coverage plot uses exclusively compatible read pairs aligned uniquely over their quasi-entire length (>80% of each read). A separate plot using only the non-unique alignments complements it. The genome is partitioned, upon projection of transcripts from RefSeq, AceView, Gencode, and iteratively of the new transcriptome derived from RNA-seq, into protein coding regions, non-coding parts of exons (UTR or non-coding transcript) and introns, giving priority to exonic coding (now 1.8% of the genome) over exonic non-coding (6.9%) over intronic (50.1%) over intergenic (defined as the remaining 41.2% of the genome, and used to compute the background noise in each run). This partition takes into account the fact that strand information was not preserved by the Illumina protocol used in this project, and the extent of the sectors would be different for a stranded protocol. The profile of coverage of each sector is computed for each sample, and the profile of the whole cohort is detailed in

Figure S2. The average coverage for intergenic, intronic and exonic (distinguishing RefSeq, AceView or Gencode which are of similar order, and the new exons) is shown in Figure SN5.

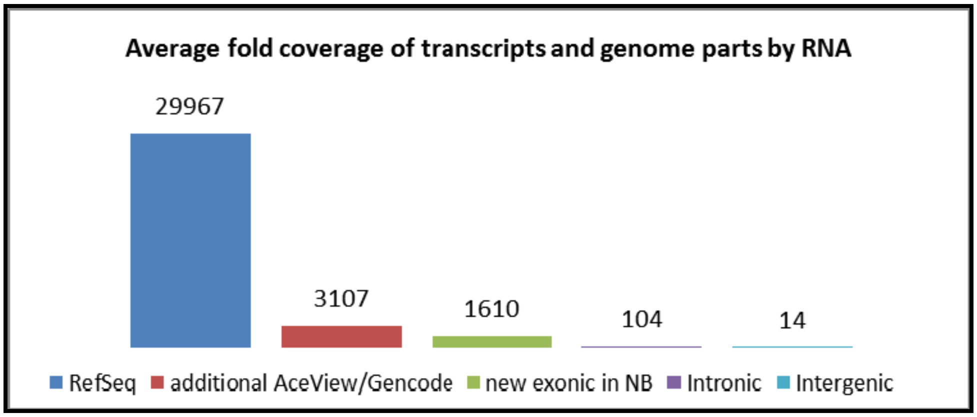

Figure SN5. *Average coverage per exonic, intronic and intergenic regions in the neuroblastoma data.*

Candidate new exons are defined in two ways: some are identified from the main coverage plot as regions with no overlap with annotated exons and covered at least 300 times in the cohort, or at least 50 times in at least 2 individuals. Other candidates may be covered at a lesser level and have overlap with previous exons but different boundaries; they are driven by new exon junctions with quantitatively compatible bordering exons (coverage of the exon junction and of the exons on both sides have to be commensurate, and compatible with an eventual sharing of coverage between variants running across the local graph). Only about one quarter of the candidate new exons and new exons-junctions are validated upon realignment and reconstruction of consistent new transcripts representing locally more than 0.5% of the previously annotated transcripts.

SUPPLEMENTARY NOTE 2: Comparative analysis of differential gene expression

Data generated by RNA-seq and microarrays were used to investigate the differential gene expression of four major clinico-genetic neuroblastoma patient subgroups (Table S1); furthermore a standard methodology and an original approach of the magic pipeline were compared.

SN2.1 Quality assessment of the microarray design

To identify comparable measures between RNA-seq and microarrays, we established an unambiguous correspondence between the 60-mer Agilent probes and the transcripts and genes. The 43,291 probes from the University of Cologne microarray were mapped to the AceView 2011 and RefSeq transcripts and to the genome as if they were RNA-seq reads. To match the Agilent design, the stranded mode was used. A validated set of 34,558 probes (79.8% of the 44 K array probes) specifically measuring a single AceView gene each – altogether 21,101 genes, 89,615 transcripts – was used for subsequent analyses (mapping file Agilent44kToAceView2011 available on the AceView website >downloads >Human >Microarray). These probes align over an average of 59.78 bases out of 60 (range: 35 to 60), to the sense strand of a unique AceView gene and to 1 to 30 transcripts each (average 3.5 transcripts), with an average of 0.048 mismatches (range 0 to 4) relative to the reference sequence. 94% of the probes have a perfect and complete match. 4,077 are designed across exon junctions (11.8% of this validated set) ensuring specific hybridization to cDNA rather than genome and often measuring alternative splice variants. 651 probes (1.9%) mapping to a gene and to an untranscribed, unannotated site on the genome (often a pseudogene) were kept as unique in the gene.

Details on the 8,733 rejected probes:

- 552 probes (1.3%) could not be mapped at the required quality
- Another 8,181 (18.9%) probes mapped well, but could not be used:
    - 6% (2,614) mapped to a single gene, but to the antisense strand. We evaluated those carefully (Figure C12) and concluded that antisense probes do not accurately measure the gene. This is why 1,580 probes mapping to a single genomic site, but to two genes in antisense, were kept in the validated set.
    - 2.0% (873) mapped well but to multiple genes (2 to 9); those ambiguous probes were dropped.
    - 9.8% (4,239 probes) mapped to a unique site on the genome, but with no annotated AceView gene
    - 1.0% (455) mapped to multiple genomic sites (2 to 9), all devoid of genes

Each of the 21,101 genes tested on the Agilent array is measured on average by 1.64 probes per gene. This redundancy is good as it often consolidates differential observations, may specifically inform on alternative variants or palliate reduced sensitivity of some probes.

SN2.2 Methodology for differential expression: MAQC-I standard approach

This approach relied on gene-level, Magic-aligned read count tables and made use of a standard 'Reads Per Kilobase per Million mapped reads' (RPKM) methodology for normalization; subsequently RPKM

values were transformed to the log_2 scale. To avoid infinite values, a constant of one (1) was added to the read counts of each gene before RPKM normalization.

For this comparative approach, RNA-seq datasets were also filtered, restricting the DE analysis to those 21,101 genes which were also represented by the microarray design. The microarray data were preprocessed and normalized according to the previously published MAQC standard methodology [6, 7].

Differentially expressed genes (DEGs) were identified according to recommendations of the MAQC-I project with a fold change (FC) ranking and a nonstringent p cutoff of 0.05 [5-8]. This simple method has been shown to yield reproducible lists of DEGs. The DEG analysis was performed using R [9]. In the per-stage comparison in this study, DEGs were identified based on the combination of cutoff values of a FC ≥ 1.5 (or ≤ 0.67) and a p-value < 0.05.

SN2.3 Methodology for differential expression: Magic pipeline approach

SN2.3a Microarray preprocessing: correlating MA to RNAseq expression data

For this analysis, the correlation between RNA-seq and Agilent microarrays was optimized by multiplicative normalization of each microarray, such that the log_2 of the signal of the $8{,}000^{th}$ most expressed probe was set to 14.5. This quantile centering regularized the Agilent data better than median centering (originally distributed), while the value 14.5 yielded a good fit to Magic RNA-seq indices.

Agilent and RNA-seq measures are poorly correlated (R around 0.5), but a more informative procedure consists in subtracting, for each gene and each probe, the average expression measured across the population, and then comparing the variations of expression relative to the average. This relative method is more sensitive than a direct correlation of indices because it is not dominated by the gene dynamics or the difference between platforms.

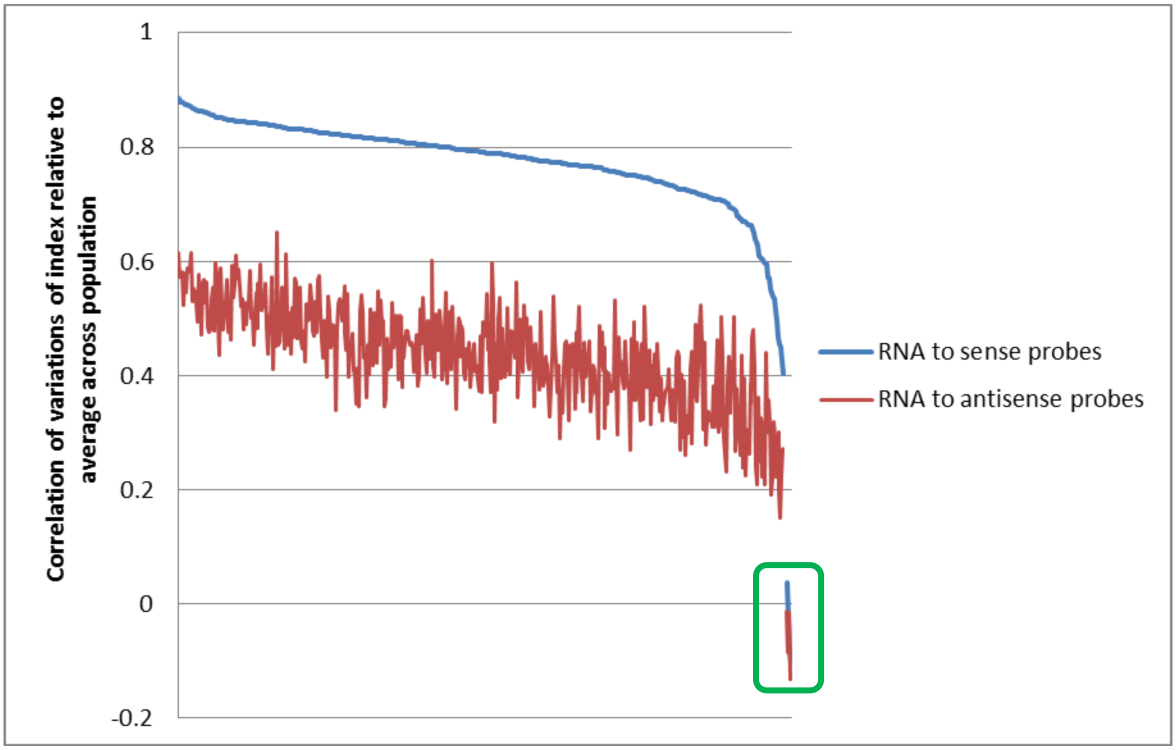

Figure SN6: *Correlation between average-subtracted expression for Agilent probes and RNA-seq for 11,254 well expressed genes measured on the array: the correlation coefficient (y axis) is measured in the 498 samples (x axis, by decreasing correlation) plus two pairs of artificially name-swapped samples (green box). It is significantly higher when measured over the 30,686 sense probes (average 0.78) than over the 2,614 antisense probes (average 0.44), showing that the Agilent protocol is mainly strand specific. Notice that under-annotation of overlapping regions in genes, or secondary hybridization in Agilent would equally well explain why the correlation for antisense probes is not null.*

To correlate Agilent arrays to RNA-seq measures, we consider the 30,686 probes forward and 2,614 reverse probes that align to a single gene over 60 bases. Then for this comparison we limit to the 11,254 genes well expressed (average index above 10) in both microarray and RNA-seq, and their probes. The relative method yields a good correlation for 90% of the samples (R = 0.8 +- 0.1); however 10% of the samples, usually from the last batch of microarrays or the last batch of RNA-seq, correlate less well (down to 0.4) (Figure SN6).

The relative method should also detect with precision any eventual sample mislabeling between Agilent and RNA-seq. As a proof of concept, two pairs of samples in AGL: Rhs955/1200 and Rhs1124/1210 were switched. For these four samples, the correlation drops around 0 (green box). As no other sample has correlation below 0.4, we conclude that no sample mislabeling occurred across the 498 samples.

We finally observe that reverse probes are less well correlated to RNA-seq (R ~ 0.3 below the sense probes), indicating that the Agilent hybridization protocol indeed preserves the strand. We therefore dropped antisense probes in subsequent analyses.

SN2.3b DE methodology: the MAGIC pipeline approach for the identification of significant differentially expressed features - DE estimated from RNA-seq and microarray data and estimation of FDR

Selection of differentially expressed genes (DEG), transcript variants (DET), exons junctions (DEJ), or microarray probes (DEP) between two conditions represented by two groups of samples G1 (in zone A in Figure SN7) and G2 (in zone B) is delicate.

1. The method needs to robustly recognize differential features, including those expressed in only one condition, to be resilient to outliers, and to be insensitive to the precise normalization of the low expressed genes, which is somewhat arbitrary on both platforms.
2. When sampling a natural population, individuals may be heterogeneous yielding multimodal distributions, for instance due to functional redundancy as there are multiple ways to achieve the same phenotype. This variability makes statistics relying too heavily on Gaussian assumptions over-optimistic, especially when evaluating p-values.
3. A conservative estimation of significance and false discovery rate is needed.

Examining for each gene (or transcript or probe or SNP or other feature) the distribution of expression indices across two groups to be compared (Figure SN7), the general idea is to delineate for each gene the eventual existence of two distinct zones of expression, the A and B zones, where either G1 or G2 predominates. For the most discriminative differentially expressed features (DEx), the G1 and G2 zones are fully separated. But in general, the two distributions overlap, leaving a gray zone in the center (Figure SN7).

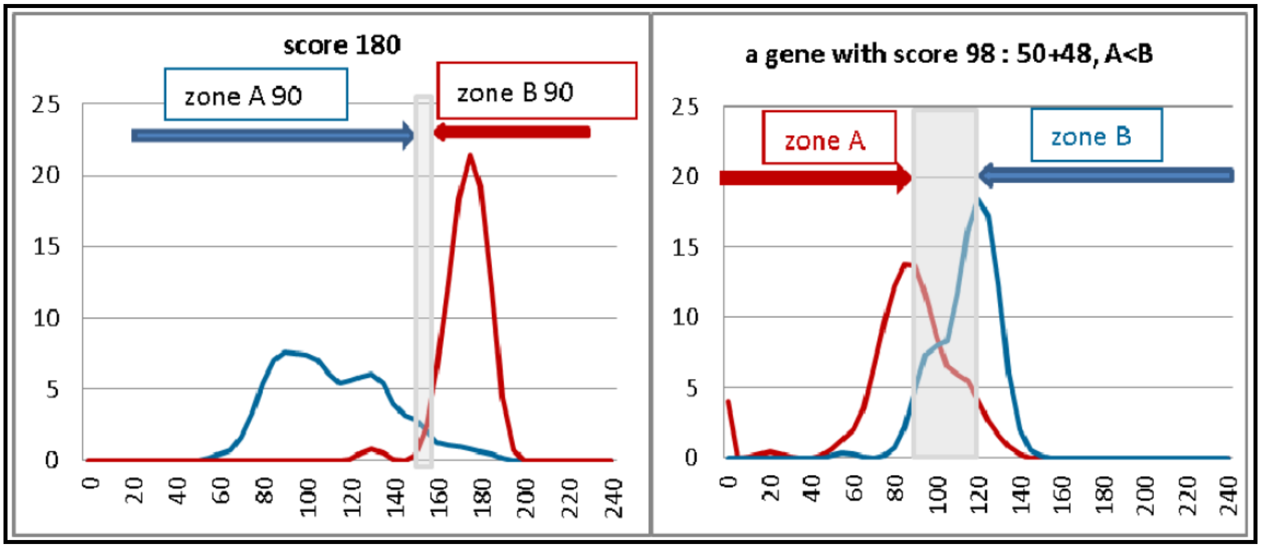

Figure SN7. *Selection of differentially expressed genes between two groups, red and blue: for each gene or feature, the normalized distribution of all individuals in each group (y axis) is plotted according to their log-based index of expression x10 (x axis). A cumulated count, starting from the extremities of the distribution, identifies the zones of dominance of the red or the blue. The choice of the purity ratio (here set at 3) allows defining the less informative (gray) index zone, where neither predominates. This method is insensitive to the exact measure of low expressors and to outliers as it cumulates with equal voting power all individuals to each side of the gray zone. The left panel shows a quasi-perfect DEG overexpressed in red, with a bimodal distribution; differential score for this gene is 90+90, most of the index range is discriminative. The right panel shows a gene overexpressed in blue and with overlapping distributions (score 50+48): this gene has discriminative power in about half the population.*

In practice, we construct the smoothed histogram of the index of expression of each feature for both groups. Each individual contributes an elemental Gaussian of area 1 and sigma 0.5 centered on the measured value. This width is not supposed to catch the sampling error, but rather to represent some biological incertitude in the measure. Both histograms are normalized to a global area of 100. We then select a heuristic 'purity ratio' R which controls the width of the gray zone (usually between 1 and 3; for example, with R=3, 75% individuals in zone A belong to G1). To locate the gray zone boundaries, we start from the left of the distribution, and count the numbers a and b of G1 and G2 individuals up to a moving frontier, and stop when (a-b) is maximal, (b-a) is then also maximal on the right. We then extend the gray zone around this frontier until on the A side, a>Rb and on the B side, b>Ra.

The differential scores are measured on each side of the gray zone as the percentage of G1 samples minus G2 samples in the A zone, and as the percentage of G2-G1 in the B zone. The sum of the A and B scores is the differential score of the gene or feature. It varies from 0 to 200, a perfect score of 100+100 indicates a DEG for which the 2 distributions do not overlap. A score of 120 = 30 + 90 would indicate that about 30% of A and 90% of B samples are classifying. For R=1, our differential score could be written as 200 (TP.TN – FP.FN)/(TP+FN).(TN+FP).

We add two conditions: the low limit of the high zone must be at least 2 fold above the average 'significantly expressed' index in the group (average 7.8, range 7 to 9 depending on the sequencing depth of the run), and minimal difference of average index between the G1 and G2 distributions was chosen to be greater than 0.4 (1.3 fold) in the NB project. Also we used R=1 and minimal gray zone >0.1. To our surprise, the 1.3 fold change yielded better agreement between RNA-seq and microarrays than demanding 2 -fold, indicating higher than usual sensitivity in differential measurements.

This simple method mostly depends on the qualitative ordering of the samples by their index of expression. It is robust against non-Gaussian distributions. It is general and was applied it to the Magic expression index defined above, but also to the Agilent microarray expression measurements, in particular for each of four clinically neuroblastoma subgroups (all *MYCN*-amplified patients, non-amplified patients with stage 1, 4, or 4S tumors) in a pairwise comparison of one subgroup versus the other three (Figure SN8).

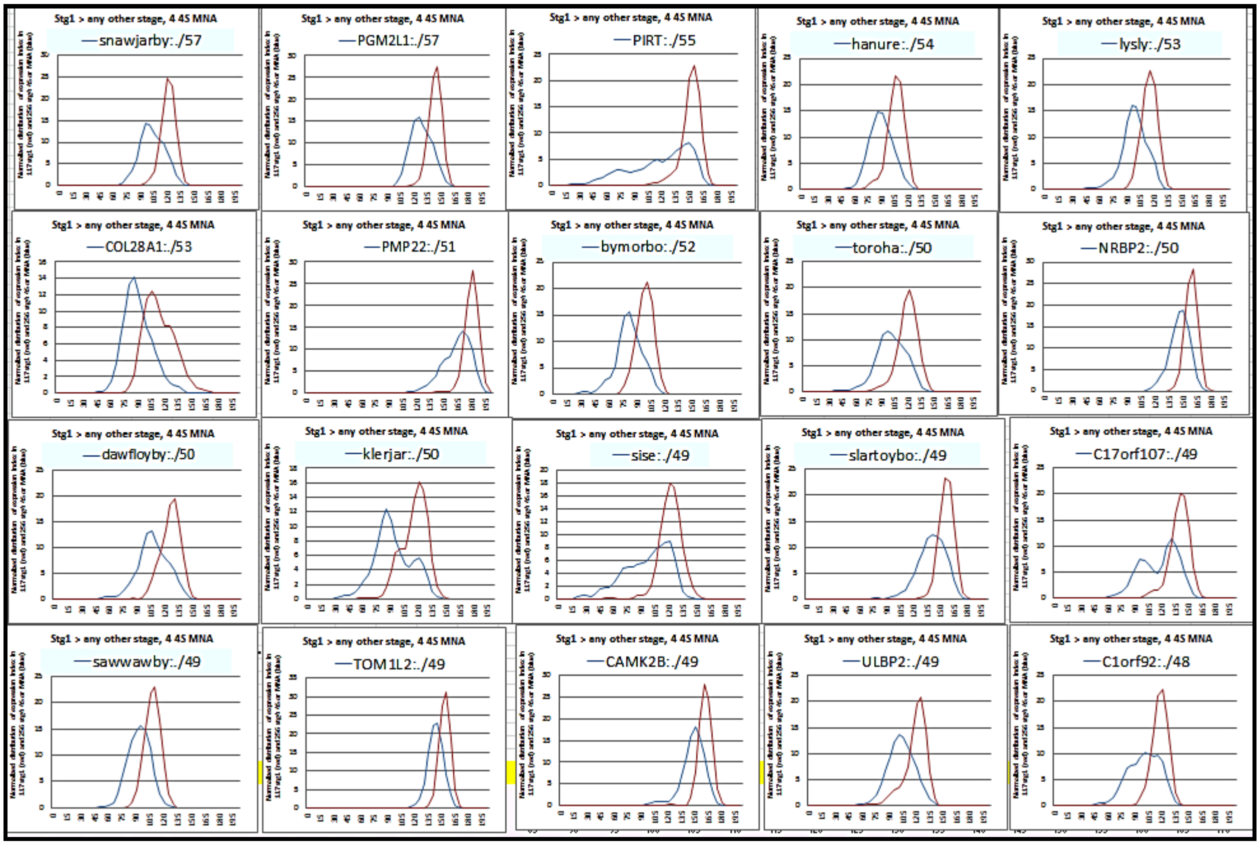

Figure SN8. *A sample of distributions of expression index in two groups analyzed to identify DEGs. For each gene, the normalized distribution of all individuals in each group (y axis) is plotted according to their log-based index of expression x10 (x axis). This sample shows the 20 top differential genes over-expressed in individuals at stage 1 without MYCN amplification (red) compared to other stages grouped (4, 4S or with MYCN amplification; blue distribution). The half score, between 57 and 48, is indicated near the gene name. Gene names highlighted in blue are AceView only, they typically represent a sizeable proportion of the top differential genes (here half).*

Because the number of tested elements is extremely large (e.g. close to 300,000 transcripts), some differential features are expected to appear at random. The false discovery rate (FDR) is estimated in Magic by resampling many times the actual experimental data. Given 2 phenotypic groups A and B of size m and n, the A+B cohort is randomly partitioned into 2 random strata, also of size m and n, both containing the same proportion m/n of A and B elements. In each resampling, the differential features, now randomized with respect to the phenotype, are called using the exact same protocol as in the real experiment. The false discovery rate is then evaluated by comparing the distribution h(s) of the differential scores between A and B (experiment) to the average k(s) of the distributions of the randomized strata (control, or noise) (Figure SN9). In practice, we perform 40 iterations at the lowest level of the C-code, when all the data are already positioned in memory, allowing fast processing (around 1 second per iteration using 70 million transcripts expression values on a single processor).

$$FDR(t) = K(t) / (H(t) + K(t)), \quad \text{with } H(t) = \sum_{s=t}^{200} h(s); \quad K(t) = \sum_{s=t}^{200} k(s)$$

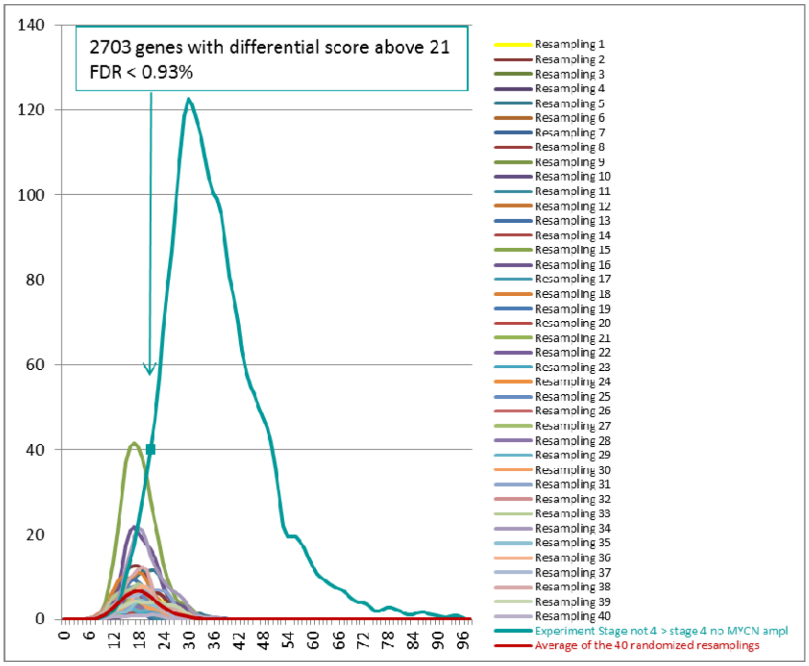

Figure SN9. *Example of differential score selection and computation of associated false discovery rate. The distribution of candidate differential genes (y axis) as a function of their differential score (x axis) is computed in the experiment (here stage 4 > non 4, blue, signal), in 40 random resampling and in their average (red), representing (systematic) noise.*

This approach allows selection of a threshold for the differential score (green arrow) adapted to the experiment and above which the differential features are significant with at most the measured FDR. It also allows a fine grain feature-by-feature correction: for instance, consider a highly differential gene in the cohort, such as XIST (a marker for sex), even a slight sex imbalance in the random strata will make it appear slightly differential. Subtracting for each differential feature its average score in the random strata from the experiment score improves the significance of the final list. Interestingly, the method is applicable even if the classes A and B are of different size (m > n) and even if the random strata are not equalized relative to external characteristics (propensity sampling). In the worst case, the number of differential elements in the random strata is increased, leading to an overestimation of the false discovery rate.

This method was evaluated in a direct comparison for determining genes as DE features and filtering on genes represented by the microarray design. In a direct comparison: DEGs of either platform were compared to the above described standard MAQC-I approach (part 2 of this Supplementary Note; see also Figure S5).

In further approach we chose to consider the increased potential of RNA-seq in detecting the entire transcriptome (unrestricted to the MA design) on a transcript-level resolution. Transcripts were chosen as DE features, i.e. a DEG via RNA-seq was defined as gene with at least one DET per stage-wise comparison, and analogous to DEGs defined by the microarray analysis (at least one DE probe defines a DEG). The resulting overall number of DEGs occurring in any of the stage-wise comparisons is given in Figure 1C.

SUPPLEMENTARY NOTE 3: *Generation of Prediction Models*

We followed the MAQC-II approach [8] to assess the influence of expression pipelines (Figure 2A) on prediction of six clinical endpoints (Table 2). The following part contains concise descriptions of each of the six data analysis teams' approaches for the generation of prediction models. Each prediction model was independently generated and evaluated. Further details of model generation are available at http://pgx.fudan.edu.cn/seqc_nb/.

Data analysis team 01

The following steps were applied for predictive modeling: (1) Five-fold cross validation for ten iterations on the six endpoints using the training data sets was performed. (2) Six categories of predictive modeling algorithms were applied for classification: K-nearest neighbor, nearest centroid, discriminant analysis, diagonal discriminant analysis, partial least squares, and logistic regression. (3) Up to 20 variable groups with sizes ranging from 10 to 100 were selected based on best ANOVA p-value and each set of variables was tested in all prediction algorithms, resulting in 100 to 120 prediction modeling conducted for each condition. (4) Cross-validation: areas under the curve (AUC) was used to select the best performing prediction model for correctly predicting the positive outcomes in each end point and the final model for each endpoint/dataset combination was applied to the validation set of the same combination. All of the above processes were performed using Partek Genomics Suite (version 6.6, release 6.12.0531, http://www.partek.com/partekgs).

Data analysis team 02

We analyzed the neuroblastoma datasets using cross validation with a suite of 84 predictive models. Basically, each model typically included some form of initial feature reduction, in which t-statistics were calculated for variable selection and the most significant features selected individually (this step was repeated separately for each cross-validation step to avoid overfitting bias). The resulting features were then used as input to a specific model drawn from one of the following general classes of algorithms: discriminant analysis, distance scoring, k-nearest neighbors, logistic regression, general linear model, partial least squares, partition trees, and radial basis machine. Each of these models was fit using a corresponding SAS procedure. To evaluate the predictive performance of the statistical classification models, 10 iterations of 5-fold cross-validation were used. Each iteration of the cross-validation loop consisted of a random partition of all of the data into five groups with each group held out in turn. The performance metrics of the statistical classification models included accuracy, the area under the curve (AUC) of the receiver operating characteristic (ROC) and the average root mean square error (RMSE). The best model for each of the endpoints was determined based on the average of these performance metrics. The best model was fitted to the full data set at the end in order to score the blinded samples for external validation performance. We used the Cross Validation Model Comparison routine in JMP Genomics software for all calculations.

Data analysis team 03

Models were computed by a predictive pipeline based on a stratified 10x 5-fold cross-validation (CV) schema and the Linear Support Vector Machines (LSVMs) with either L1 or L2 for both penalty and loss function ([10], http://www.csie.ntu.edu.tw/~cjlin/liblinear). In each internal training set, variables were rescaled to [-1, 1] and the optimal LSVM regularization parameter (C_{opt}) was chosen among a grid (C_GRID) by maximizing the average Matthews Correlation Coefficient (MCC) computed in a 10x stratified random subsampling CV (50-50% training-test proportion). Features were ranked according to the square of model weights. C_GRID we selected were 0.00001, 0.0001, 0.001, 0.01, 0.1, 1, 10, 100, 1000, and 10000, and FSTEPS we used were 1, 2, 3, 4, 5, 6, 7, 8, 9, 10, 20, 30, 40, 50, 60, 70, 80, 90, 100, 200, 300, 400, 500, 600, 700, 800, 900, 1000, 2000, 3000, 4000, 5000, 6000, 7000, 8000, 9000 and 10000. In each internal validation set, variables were rescaled according to the parameters of the internal training set and a series of LSVM models, built upon an increasing number of the ranked features, were evaluated in terms of MCC. The average MCC curve over the 10x5 CV iterations was computed for the different feature set sizes, together with a unified feature list ranked by average positions [11]. The feature set size maximizing the average MCC was finally selected. The machine learning steps were built on top of the MLPY Python package ([12]; http://mlpy.sourceforge.net).

Data analysis team 04

We used a grid search procedure which searched through various trade-offs of mean difference test and t-test. For any pair of thresholds, the features that satisfied both thresholds were used to build a diagonal linear discriminate analysis (*DLDA*) classifier [13], which was a simple linear classifier similar to weighted voting [14]. By varying the thresholds of these two statistics in predefined steps, we could achieve various trade-offs and control the size of the feature sets. The pair of the thresholds of the two statistics solely determined the feature set and thus the *DLDA* model, as there was no parameter to be tuned for *DLDA* modelling. The final prediction model was generated using the optimal trade-off in terms of cross-validated model performance. We used area of the ROC (receiver operating characteristic) curve (AUC) to measure model performance. All of the above procedures were implemented in an in-house Java package *Array Data Analyzer* [15].

Data analysis team 05

The best models were obtained using 10x 5-fold cross-validation method and the linear support vector machines algorithm (http://www.csie.ntu.edu.tw/~cjlin/liblinear/). In summary, our approach proceeded in two phases. (1) Feature reduction: in each cross-validation step, wilcoxon sum rank test was performed and features were ranked by p value, the top 1000 most significant features were selected. Next, the support vector machine recursive feature extraction (SVM-RFE) algorithm was implemented to reorder the selected features, and then the top 100 features were selected as input of next step. (2) Feature selection: the SVM model was trained on the internal training set and a series of models were built by choosing different feature set sites (1, 2, 3, 4…100) with an increasing number of the ranked 100 features. Two SVM parameters were chosen among a grid (gamma=10^(-6:0),cost=c(1,5,10,100)) by maximizing the Matthews Correlation Coefficient (MCC). For one cross-validation step, The MCC was

calculated for each model, and the feature set size maximizing the MCC was selected as a best model. Finally, we got 50 best models. Then the frequency of each feature in all the 50 models was calculated, features involved in at least 10 best models were selected as the best model for prediction of validation data set.

Data analysis team 06

We performed student's t-tests on each endpoint of the neuroblastoma datasets, and used the resulting p-values along with a fold-change cutoff to limit the number of features to only those features that exhibit high significance. To determine the most usable features for classification, five expression profiles (MAV_G, MAV_T, TAV_G, TUC_G, and TUC_T) applied feature selection methods like info-gain, gain ratio, SVMattribuEval and CfsSubsetEval in Weka [16-18], with a 10-fold cross-validation to limit overfitting. When training with the selected features, we used a suite of classification methods: libSVM, Neuronetwork, SMO, logistic, randomforest, and random tree, to build a set of models, then selected the models which consistently exhibited the best sensitivity/specificity/AUC results. The remaining four expression profiles (MAV_J, TAV_T , TAV_J, and TUC_J) used SAS JMP Genomics' model comparison system [19], instead of Weka, to calculate a similar set of models based on the following classification models: Generalized Linear Model, Partial Least Squres, and Logistic Regression, with a 5-fold cross-validation.

REFERENCES

1. Lenos K, Grawenda AM, Lodder K, Kuijjer ML, Teunisse AF, Repapi E, Grochola LF, Bartel F, Hogendoorn PC, Wuerl P, et al: **Alternate splicing of the p53 inhibitor HDMX offers a superior prognostic biomarker than p53 mutation in human cancer.** *Cancer Res* 2012, **72:**4074-4084.
2. Nishi T, Lee PS, Oka K, Levin VA, Tanase S, Morino Y, Saya H: **Differential expression of two types of the neurofibromatosis type 1 (NF1) gene transcripts related to neuronal differentiation.** *Oncogene* 1991, **6:**1555-1559.
3. Wang C, Gong B, Bushel PR, Thierry-Mieg J, Thierry-Mieg D, Xu J, Fang H, Hong H, Shen J, Su Z, et al: **The concordance between RNA-seq and microarray data depends on chemical treatment and transcript abundance.** *Nat Biotechnol* 2014, **32:**926-932.
4. Peng X, Thierry-Mieg J, Thierry-Mieg D, Nishida A, Pipes L, Bozinoski M, Thomas MJ, Kelly S, Weiss JM, Raveendran M, et al: **Tissue-specific transcriptome sequencing analysis expands the non-human primate reference transcriptome resource (NHPRTR).** *Nucleic Acids Res* 2015, **43:**D737-742.
5. Su Z, Łabaj PP, Li S, Thierry-Mieg J, Thierry-Mieg D, Shi W, Wang C, Schroth GP, Setterquist RA, Thompson JF, et al: **A comprehensive assessment of RNA-seq accuracy, reproducibility and information content by the Sequencing Quality Control Consortium.** *Nat Biotech* 2014, **32:**903-914.
6. Guo L, Lobenhofer EK, Wang C, Shippy R, Harris SC, Zhang L, Mei N, Chen T, Herman D, Goodsaid FM, et al: **Rat toxicogenomic study reveals analytical consistency across microarray platforms.** *Nat Biotechnol* 2006, **24:**1162-1169.
7. Shi L, Reid LH, Jones WD, Shippy R, Warrington JA, Baker SC, Collins PJ, de Longueville F, Kawasaki ES, Lee KY, et al: **The MicroArray Quality Control (MAQC) project shows inter- and intraplatform reproducibility of gene expression measurements.** *Nat Biotechnol* 2006, **24:**1151-1161.
8. Shi L, Campbell G, Jones WD, Campagne F, Wen Z, Walker SJ, Su Z, Chu TM, Goodsaid FM, Pusztai L, et al: **The MicroArray Quality Control (MAQC)-II study of common practices for the development and validation of microarray-based predictive models.** *Nat Biotechnol* 2010, **28:**827-838.
9. R Core Team: **R: A language and environment for statistical computing. .** Vienna, Austria: R Foundation for Statistical Computing. ; 2012.
10. Fan R-E, Chang K-W, Hsieh C-J, Wang X-R, Lin C-J: **LIBLINEAR: A library for large linear classification.** *The Journal of Machine Learning Research* 2008, **9:**1871-1874.
11. Jurman G, Merler S, Barla A, Paoli S, Galea A, Furlanello C: **Algebraic stability indicators for ranked lists in molecular profiling.** *Bioinformatics* 2008, **24:**258-264.
12. Albanese D, Visintainer R, Merler S, Riccadonna S, Jurman G, Furlanello C: **mlpy: Machine learning Python.** *arXiv preprint arXiv:12026548* 2012.
13. Dudoit S, Fridlyand J, Speed TP: **Comparison of discrimination methods for the classification of tumors using gene expression data.** *Journal of the American statistical association* 2002, **97:**77-87.
14. Golub TR, Slonim DK, Tamayo P, Huard C, Gaasenbeek M, Mesirov JP, Coller H, Loh ML, Downing JR, Caligiuri MA, et al: **Molecular classification of cancer: class discovery and class prediction by gene expression monitoring.** *Science* 1999, **286:**531-537.
15. Cheng J, Greshock J, Shi L, Painter J, Lin X, Lee K, Zheng S, Wooster R, Pusztai L, Menius A: **An adaptive feature selection method for microarray data analysis.** In *Bioinformatics and Biomedicine (BIBM), 2012 IEEE International Conference on*. IEEE; 2012: 1-6.
16. Hall M, Frank E, Holmes G, Pfahringer B, Reutemann P, Witten IH: **The WEKA data mining software: an update.** *ACM SIGKDD explorations newsletter* 2009, **11:**10-18.

17. Hall MA: **Correlation-based feature selection for machine learning.** The University of Waikato, 1999.
18. Weston J, Mukherjee S, Chapelle O, Pontil M, Poggio T, Vapnik V: **Feature Selection for SVMs.** http://www0.cs.ucl.ac.uk/staff/M.Pontil/reading/featsel.pdf.
19. SAS Institute Inc.: **JMP® 9 Modeling and Multivariate Methods.** Cary, NC: SAS Institute Inc.; 2010.
